# Vertical and horizontal gene transfer tradeoffs direct plasmid fitness

**DOI:** 10.15252/msb.202211300

**Published:** 2022-12-27

**Authors:** Jonathan H Bethke, Helena R Ma, Ryan Tsoi, Li Cheng, Minfeng Xiao, Lingchong You

**Affiliations:** ^1^ Department of Molecular Genetics and Microbiology Duke University NC Durham USA; ^2^ Department of Biomedical Engineering Duke University NC Durham USA; ^3^ Center for Quantitative Biodesign Duke University NC Durham USA; ^4^ BGI‐Shenzhen Shenzhen China; ^5^ Shenzhen Key Laboratory of Unknown Pathogen Identification, BGI‐Shenzhen Shenzhen China; ^6^ School of Biology and Biological Engineering South China University of Technology Guangzhou China

**Keywords:** conjugation, fitness, plasmid, resistance, tradeoff, Genetics, Gene Therapy & Genetic Disease, Microbiology, Virology & Host Pathogen Interaction

## Abstract

Plasmid fitness is directed by two orthogonal processes—vertical transfer through cell division and horizontal transfer through conjugation. When considered individually, improvements in either mode of transfer can promote how well a plasmid spreads and persists. Together, however, the metabolic cost of conjugation could create a tradeoff that constrains plasmid evolution. Here, we present evidence for the presence, consequences, and molecular basis of a conjugation‐growth tradeoff across 40 plasmids derived from clinical *Escherichia coli* pathogens. We discover that most plasmids operate below a conjugation efficiency threshold for major growth effects, indicating strong natural selection for vertical transfer. Below this threshold, *E. coli* demonstrates a remarkable growth tolerance to over four orders of magnitude change in conjugation efficiency. This tolerance fades as nutrients become scarce and horizontal transfer attracts a greater share of host resources. Our results provide insight into evolutionary constraints directing plasmid fitness and strategies to combat the spread of antibiotic resistance.

## Graphical abstract

Quantification of natural plasmid transfer rates reveals a tradeoff between conjugation and cell division that constrains plasmid evolution.

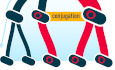

## Introduction

**Figure 1:**
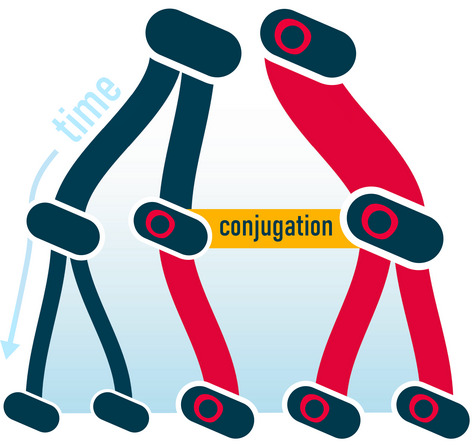
Vertical and horizontal plasmid transfer In vertical transfer, cells (dark blue) divide and generate copies of themselves. Faster growth produces more cells over time. Plasmids (red) may slow growth but may also horizontally transfer to plasmid‐free cells in a process called conjugation (yellow). Together, these processes outline plasmid fitness or how well a plasmid spreads.

Plasmids are ubiquitous and integral agents of bacterial evolution, demonstrated by many pathogens' acquisition of antibiotic resistance genes. The fitness of a plasmid, or the degree to which it is passed on over time, is directed by vertical and horizontal transfer (Fig 1). Vertical transfer refers to the passage of a plasmid from mother to daughter cells during division. Horizontal transfer refers to the passage of a plasmid from donor to any recipient cell outside of cell division, often through conjugation (Smillie *et al*, 2010; Rodríguez‐Beltrán *et al*, 2021). In the absence of positive selection, plasmids can reduce host growth such that plasmid‐carrying cells will be outcompeted by plasmid‐free cells through vertical transfer (Alonso‐del Valle *et al*, 2021). This disadvantage can be reduced by compensatory evolution (Loftie‐Eaton *et al*, 2017), co‐selection (Andersson & Hughes, 2011), host variety (Porse *et al*, 2016; Alonso‐del Valle *et al*, 2021), and high rates of conjugation (Lopatkin *et al*, 2017). As a result, plasmids can survive and spread in microbial communities even in the absence of positive selection.

Individually, these processes promote plasmid stability; however, the metabolic cost of conjugation may slow host growth rates. Conjugation is a complex process that can occur in rapid succession, requiring upwards of 30 transfer (*tra*) genes and the replicative transfer of plasmid DNA (de la Cruz *et al*, 2010; San Millan & MacLean, 2017). This metabolic investment associated with conjugation could create a tradeoff between vertical and horizontal plasmid transfer: a low metabolic cost facilitates cell division but limits conjugation, whereas high conjugation efficiency spreads to new cells but limits their division. In this way, the evolution of plasmid transfer could be constrained.

Tradeoffs have long been hypothesized in the vertical and horizontal transfer of parasites to better understand and control their spread (Anderson & May, 1982; Turner *et al*, 1998; Hall *et al*, 2017). They suggest an optimal balance exists between transfer modes, dependent on the reproductive value of each. Knowledge of fitness optima allows evolutionary prediction and intervention by changing the reproductive value of vertical or horizontal transfer. Despite plasmids' major role in bacterial evolution, the tradeoff interaction of conjugation and growth has received little attention. Among the few studies on the interaction, evidence for the tradeoff is inconsistent and limited involving small numbers of plasmids evolved in laboratory settings (Turner *et al*, 1998; Dahlberg & Chao, 2003; Kottara *et al*, 2016; Dimitriu *et al*, 2021; Hall *et al*, 2021). Furthermore, the growth effects associated with plasmid carriage are increasingly attributed to specific plasmid–chromosome interactions over more general traits like conjugation efficiency (Loftie‐Eaton *et al*, 2017; San Millan *et al*, 2018; Hall *et al*, 2021; Rodríguez‐Beltrán *et al*, 2022). This leaves the generality, consequences, and molecular basis of a conjugation‐growth tradeoff in question.

Here, we integrate genomics, transcriptomics, and phenomics to assess conjugation‐growth tradeoffs across 40 diverse plasmids from clinical *E. coli* pathogens. Our plasmid‐centric approach reveals a general transfer tradeoff, where major growth effects arise past a conjugation efficiency threshold. This directs plasmid fitness at a fundamental level: higher conjugation efficiencies do not necessarily yield higher plasmid fitness. We find a standard MG1655 *E. coli* host that can support a wide range of conjugation efficiencies with only minor growth effects, placing most plasmids below the threshold. For this majority of plasmids with similar growth effects, conjugation efficiency then serves to differentiate and predict plasmid fitness. We further demonstrate a molecular basis for conjugation efficiency and its associated growth effects with practical application to the future study of plasmid biology.

## Results

### A tradeoff between vertical and horizontal gene transfer

*Escherichia coli*, an exemplar of plasmid‐mediated acquisition of antibiotic resistance, is our model for studying plasmid dynamics (Koraimann, 2018). Isolates from patient bloodstream infections at Duke University Hospital were collected from 2002 to 2014 and screened for their ability to transfer resistance against beta‐lactams, the most commonly used class of antibiotics. Among these is sequence type 131 (ST131), a pandemic clonal group responsible for up to 30% of *E. coli* infections and closely associated with incompatibility (Inc) F plasmids (Nicolas‐Chanoine *et al*, 2014; Stoesser *et al*, 2016).

In previous work, we discovered 35 of 143 (~ 25%) *E. coli* pathogens were able to readily transfer their beta‐lactam resistance to a susceptible MG1655 *E. coli* recipient (strain DA28102, Appendix Table S1; Bethke *et al*, 2020). With additional screening, we assembled the collection of 40 MG1655 transconjugants used in this study, each carrying a unique set of antimicrobial‐resistant (AMR) plasmids. A consistent MG1655 host enables general comparison of the AMR plasmids by excluding confounding host effects. Long‐read, PacBio sequencing capable of discerning the structure of mobile elements was performed for each pathogen isolate and is available at BioProjects PRJNA551684 and PRJNA823807. AMR plasmids were found to be genetically diverse, spanning multiple Inc groups, MOB relaxases, sizes, and resistances (Appendix Table S1). Follow‐up sequencing was performed to determine the plasmid composition of transconjugants. From the 27 pathogen donors carrying multiple plasmids, only 4 (15%) MG1655 transconjugants received and maintained two plasmids (Appendix Table S1). All other transconjugants carry a single, conjugative plasmid from the pathogen donor.

Plasmid growth effects (ω) on the MG1655 host were calculated as: $1-\left(\mu_{p+}/\mu_{p-}\right)$, where *μ* is the max growth rate for plasmid‐carrying (*p+*) and plasmid‐free (*p*−) strains, such that values >0 indicate burden and values <0 indicate benefit. Growth rates were measured in plate readers at OD_600_ across three environments of increasing nutrient richness: M9, M9CA, and TB (Appendix Fig S1). A strong correlation (*R* ^2^ = 0.87) was found between growth rate and area under the growth curve (Appendix Fig S2).

Conjugation efficiencies (η, (cells/ml)^−1^ h^−1^) were measured as a function of donor, recipient, and transconjugant densities after a 1 h conjugation period in liquid M9CA media at room temperature to prevent growth (Lopatkin *et al*, 2016; Bethke *et al*, 2020). Strain fAYC002 was used as a plasmid‐free MG1655 *E. coli* recipient consistent with DA28102 donors (Appendix Table S1; Chen *et al*, 2014). Preventing growth ensures accurate measurements of conjugation efficiency by disentangling growth and conjugation effects on final cell density. Isolates were grown in M9, M9CA, or TB media prior to conjugation under growth restriction conditions.

**Figure 2:**
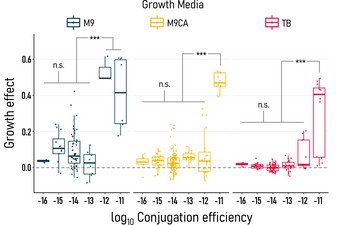
A general tradeoff between vertical and horizontal plasmid transfer MG1655 *Escherichia coli* hosts tolerate a wide range of conjugation efficiencies ((cells/ml)^−1^ h^−1^) from diverse AMR plasmids with minor growth effects. Over four orders of magnitude change in conjugation efficiency were supported with no significant change in growth burden (*P* > 0.7, Tukey HSD). Only at high conjugation efficiencies (η ≥ 10^−12^) does a growth tradeoff develop (***P < 0.001, Tukey HSD). Biological replicates for growth effect (*n* = 3–5 per strain per environment) are shown for low M9 (blue), intermediate M9CA (yellow), and high TB (red) nutrient environments, with average conjugation efficiencies (*n* = 3–6 technical replicates, 1–2 biological replicates) binned in 10‐fold increments. Boxes indicate 25^th^, 50^th^, and 75^th^ percentiles with whiskers extending to maximum and minimum values within 1.5 times the interquartile region.

Our measurements reveal that most AMR plasmids cause minor growth effects, regardless of multidrug resistances and novelty to the MG1655 *E. coli* recipient (Fig 2 and Appendix Figs S3 and S4). Conversely, conjugation efficiencies varied over six orders of magnitude (10^−16^–10^−10^ (cells/ml)^−1^ h^−1^). For subsequent reference, we divide this range into three equal subranges in log‐scale to denote low, intermediate, and high conjugation efficiencies. There was a significant effect of media environment on growth effect per strain [*F*(78, 398) = 6.27, *P* < 0.0001, ANOVA] and conjugation efficiency [*F*(1, 52) = 4.92, *P* = 0.031, ANOVA], specifically between TB and M9 media. Generally, growth effects decreased and conjugation efficiency increased with increasing nutrient content (Appendix Fig S5). Particularly in high nutrient environments, the MG1655 host displayed a surprising tolerance to changes in conjugation efficiency. Over four orders of magnitude change in conjugation efficiency were supported with no significant change in growth effect (*P* > 0.7, Tukey HSD).

However, a tradeoff emerged beyond a conjugation efficiency threshold in each nutrient environment. For plasmids past the threshold, conjugation efficiency was correlated with increasing growth burden (Fig 2). Plasmids with high conjugation efficiency imposed significantly more growth burden than all other plasmids in the same environment (*F*(2, 404) = 6.35, *P* = 0.002, ANOVA with *post hoc* Tukey HSD). As the threshold decreases with nutrient level, it is likely representative of a host's tolerance to cellular resources being diverted toward conjugation. Growth effects and conjugation efficiency collectively exhibit a linear–threshold relationship, where plasmid‐free and plasmid‐carrying growth rates do not significantly differ before the threshold (*F*(2, 335) = 0.65, *P* = 0.52, ANOVA and Appendix Fig S3). This relationship is found across genetically diverse, single AMR plasmids and therefore does not appear to be specific to plasmid compositions (Appendix Table S1 and Appendix Fig S3). Plasmid copy number may play a role, however, as estimated total plasmid DNA weakly correlates with both growth effect and conjugation efficiency (Appendix Fig S6).

### Balancing conjugation and growth

All else being equal, higher conjugation efficiency would yield higher plasmid fitness (Wang & You, 2020). However, with the tradeoff described above, intermediate conjugation efficiencies that balance growth effects may instead yield the highest plasmid fitness. As growth effects and conjugation efficiencies change with the environment, so too would the balance between them (Fig 2 and Appendix Fig S5). Using a piecewise fit between growth effects and conjugation efficiency, we modeled a two‐population system (i.e., with and without plasmid) to predict both the plasmid‐carrying fraction (% plasmid) and plasmid abundance in high and low nutrient environments (Appendix Fig S3 and Materials and Methods). Each simulation begins with a 1:1 mixture of plasmid‐free to plasmid‐carrying cells diluted 1,000‐fold from the carrying capacity. The collective fitness effects of conjugation efficiency and growth are captured in plasmid‐carrying fraction and plasmid abundance.

**Figure 3:**
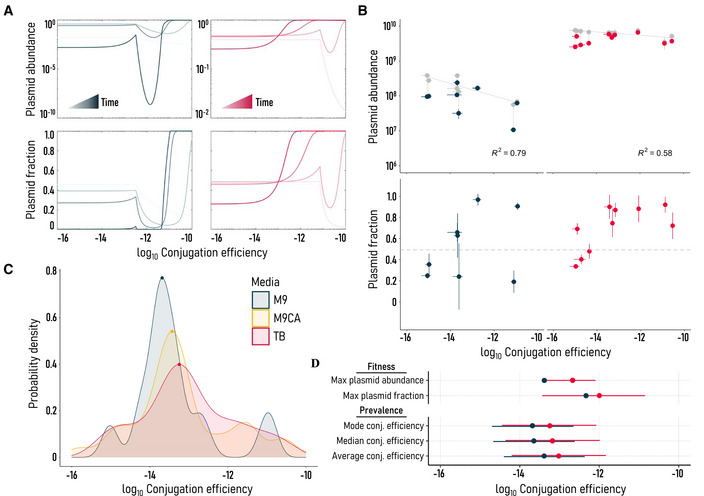
Transfer tradeoffs direct plasmid fitness Tradeoffs between conjugation efficiency and growth shape plasmid abundance and plasmid‐carrying fraction (% plasmid) in nutrient‐poor M9 (blue) and rich TB (red) simulations. Simulation results are shown for 10, 100, 1,000, and 10,000 h timepoints, indicated by increasing color saturation. In both environments, plasmid abundance and fraction peaks emerge at intermediate‐high conjugation efficiencies near the threshold for growth burden (Appendix Fig S2).Experimental AMR plasmid abundance (cells/ml) and plasmid‐carrying fraction peak at intermediate conjugation efficiencies (10^−14^ – 10^−12^ (cells/ml)^−1^ h^−1^) in nutrient‐poor M9 and rich TB environments. Total (gray) and plasmid‐carrying (blue, M9; red, TB) cell densities are linked to indicate shared culture. Plasmid‐free and plasmid‐carrying MG1655 *Escherichia coli* were diluted 1,000‐fold, mixed 1:1 (dashed line), and passaged daily for 3 days with 100,000‐fold dilutions before measuring. Each point is a unique strain representing the average of at least triplicate biological measurements ± SD. Strains tested include 41T, 92T, 94T, 2350T, 2629T, 3204T, 4219T, 4563T, 4592T, and 5696T. Conjugation efficiencies for strains 2350T and 3204T fell outside the measurable range in M9 media.Prevalence of AMR plasmid conjugation efficiencies in MG1655 *E. coli* across M9, M9CA, and TB media. Probability density distribution modes are indicated by points.Summary statistics comparing plasmid abundance and fraction maxima with conjugation efficiency prevalence in TB and M9 media. Points indicate fitted values, with bars added to reflect (i) other fitness points at or near maximum values or (ii) one standard deviation in the prevalence distribution.

Simulated plasmid abundance and plasmid‐carrying fraction are shown in Fig 3A for high and low nutrient environments. Over time, plasmid abundance and plasmid‐carrying fraction peaks emerge at both intermediate and high conjugation efficiencies. The intermediate peaks occur at the tradeoff threshold and, in the high nutrient environment, ultimately merge with the high conjugation efficiency peak as time progresses. However, higher conjugation efficiencies do not necessarily yield higher plasmid fitness.

This outcome is due to the growth, time, and recipient dependence of conjugation (Turner *et al*, 1998; Dimitriu *et al*, 2021). In the low nutrient environment, higher growth burdens and slower overall growth rates produce a plasmid abundance and fraction trough at high conjugation efficiencies (Fig 3A). High conjugation efficiency plasmids in this trough region are predicted to have the lowest fitness of all across all timepoints. This trough is also present in the high nutrient environment, but it is abolished at later time points. It takes time for high conjugation efficiency plasmids to overcome high growth burdens and reach high abundance. For this reason, high conjugation efficiencies may be outcompeted in real environments where recipient density remains low or competitor plasmids are present to inhibit conjugative spread. High conjugation efficiency plasmids are indeed rare among clinical *E. coli* pathogens and many plasmids transferred faster in the naïve *E. coli* host compared with the native pathogen host (Appendix Figs S5C and S7; Bethke *et al*, 2020).

We tested these consequences of a conjugation‐growth tradeoff experimentally for 10 AMR plasmids spanning a wide range of conjugation efficiencies (Fig 3B). For each plasmid, we recreated the starting conditions of the simulation: passaging a mixture of plasmid‐carrying and plasmid‐free MG1655 *E. coli* daily for 3 days, then measuring abundance and plasmid‐carrying fraction. Few passages and strong dilutions (100,000‐fold) were chosen to limit evolution and maximize exponential phase duration, respectively. By the end of the 3 days, all plasmids had persisted.

In agreement with the simulations, plasmid abundance and plasmid‐carrying fraction maxima are found at intermediate conjugation efficiencies (10^−14^ – 10^−12^ (cells/ml)^−1^ h^−1^) in both high and low nutrient environments (Fig 3A and B). These fitness maxima shift to lower conjugation efficiencies as nutrients decrease. Consistent with conjugation being resource‐intensive, total cell density decreased with increasing conjugation efficiency (*R* ^2^ = 0.79, M9; 0.58, TB, Fig 3B). If a conjugation‐growth tradeoff is a general phenomenon, different plasmids may approach similar conjugation efficiencies through convergent evolution. Indeed, the distributions of all conjugation efficiencies across nutrient environments reflect the fitness results in Fig 3B and C. Intermediate conjugation efficiencies that yielded the highest abundance or fraction are among the most common conjugation efficiencies across all plasmids (Fig 3D).

### From transcripts to transfers

A conjugation‐growth tradeoff across diverse *E. coli* plasmids in the same host suggests a shared, plasmid‐based mechanism. Outside of plasmid resistances (Appendix Fig S4) or composition (Appendix Fig S3), conjugative transfer (*tra*) machinery remains a constant and plausible source of growth effects. The relationship between *tra* machinery and conjugation efficiency has not been extensively quantified nor has their relationship been framed in the context of fitness consequences.

To establish the link between conjugation efficiency and *tra* machinery, we performed RNA sequencing. Gene expression was measured for MG1655 *E. coli* transconjugants ESBL41T, ESBL92T, GN02560T, GN05243T, and pCU1T carrying AMR plasmids of IncN/F, IncF/I, IncF/Col, IncB/O/K/Z, and IncN groups, respectively, a wide range of conjugation efficiencies, and varied growth effects in both TB and M9 media. All strains were prepared following our conjugation protocol, with RNA collected at the conjugation phase.

**Figure 4:**
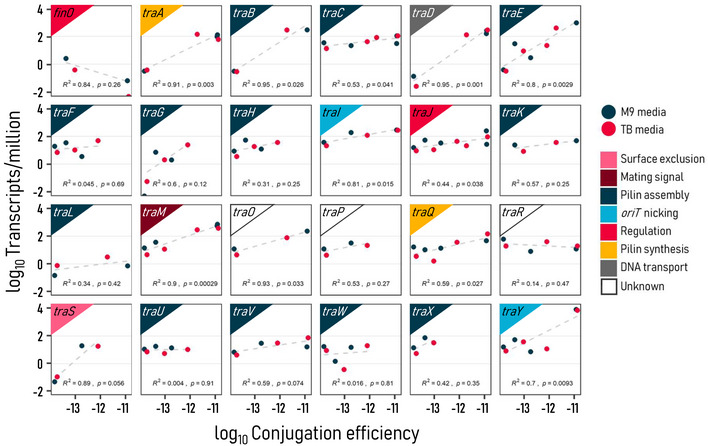
Conjugation efficiency correlates with *tra* gene expression Expression of the transfer (*tra*) gene family broadly correlates with conjugation efficiency ((cells/ml)^−1^ h^−1^) across five unique plasmids, each in the same MG1655 *E. coli* host. IncN/F, IncF/I, IncF/Col, IncB/O/K/Z, and IncN plasmids were chosen for diversity in Inc groups, conjugation efficiency, and growth effects across nutrient environments. Average transcripts per million (TPM) and η for both M9 (blue) and TB (red) media are shown from three biological replicates. Gene and gene function are indicated in the upper left. Correlation coefficients are calculated for log values as displayed.

We find a striking correlation between normalized expression, calculated as transcripts per million (TPM), and conjugation efficiency across 24 *tra* genes (Fig 4). Two genes, *traN* and *traT*, were excluded as each only had a single data point. Expression was positively correlated with conjugation efficiency for most genes, notably *traJ*, *traI*, *traM*, and *traY*, with only *finOP* showing a strong negative correlation. An opposing trend may be expected as *finOP* represses conjugation (Frost & Koraimann, 2010). It is particularly surprising that the correlation is maintained across IncN, IncN/F, IncF/I, IncF/Col, and IncB/O/K/Z groups, a wide range of conjugation efficiencies, and different nutrient environments. This robustness suggests conjugation may be quantified transcriptionally. Together, these data detail a fine‐grain relationship between conjugation and gene expression that is not readily apparent from growth effects.

### The relative cost of horizontal transfer within a host

**Figure 5:**
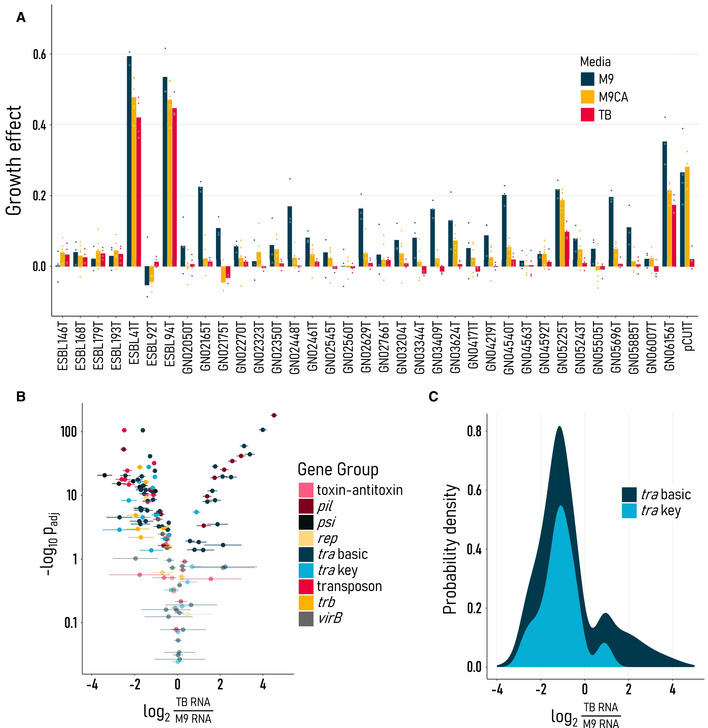
Plasmid growth effect follows horizontal gene transfer expression Per strain growth burden significantly decreases as nutrients increase, relative to media‐matched, plasmid‐free controls [*F*(70, 21) = *P* < 0.0001, ANOVA]. Bars are colored by media and represent the average of the biological replicate points shown (*n* = 3–5 per strain per environment).Differential horizontal gene transfer expression in nutrient‐rich TB and poor M9 media matches growth effect trends, favoring horizontal over vertical transfer under nutrient stress. Expression was measured in strains ESBL41T, ESBL92T, GN02560T, GN05243T, and pCU1T, and the adjusted significance (*P*_adj_) of differential expression is given on the y axis. Genes are colored by group function.Of the *tra* genes with significant differential expression, the majority have a higher relative expression in M9 than in TB media.

Whereas conjugation efficiency increases as hosts devote more resources to *tra* expression (Fig 4), growth effects may only manifest past an expression threshold (Fig 2). This poses a challenge when relating expression levels to growth effects for most plasmids below the threshold. We observed that, relative to a plasmid‐free control in each environment, plasmids almost uniformly exerted higher growth burdens on their host in nutrient‐poor M9 over rich TB media (Fig 5A). These per‐strain changes in growth effect allow broad comparison with changes in relative *tra* expression.

To this end, we determined the fraction of total transcriptional resources devoted to horizontal transfer in M9 versus TB media. Of 141 genes known to be involved in horizontal gene transfer (e.g., *tra*, *pil*, *trb* gene families), 102 (72%) displayed significant differential expression (Fig 5B and C). Of these 102 differentially expressed genes, 72 (71%) had higher relative expression in nutrient‐poor M9 rather than rich TB media, including 13 of 14 (93%) key regulators of plasmid conjugation (i.e., *traJ/I/M/Y* and *finOP*; de la Cruz *et al*, 2010; Koraimann, 2018). Most genes with significantly higher expression in TB (16/23, 70%) came from strain 92T—the only transconjugant with a higher average growth burden in TB than in M9 (Appendix Fig S8). These matching trends between growth burden and gene expression, in both positive and negative directions, offer a mechanistic basis for conjugative plasmid growth burdens. This is consistent with a recent study in which a *finO* knockout increases both conjugation efficiency and growth burden through *tra* overexpression (Rajer & Sandegren, 2022).

From an ecological standpoint, our results suggest plasmids become increasingly parasitic to their hosts when nutrients are scarce. Conjugative transfer consumed a greater share of resources in M9 media at the expense of already lowered host growth rates (Fig 5C and Appendix Fig S1C). In a stressed or dying host undergoing minimal cell division, the reproductive value of vertical transfer diminishes for a plasmid. Conversely, horizontal transfer to abundant new or healthier hosts increases in reproductive value (Turner *et al*, 1998; Dimitriu *et al*, 2021), sometimes at the expense of the current host. Examples of this behavior are seen in the infectious transmission of parasites (Anderson & May, 1982; Bull, 1994; Kaltz & Koella, 2003).

Temporarily increased horizontal transfer, as a mechanism of rapid adaptation, may also benefit hosts experiencing transient stress. This is especially applicable in slow growth environments (e.g., M9 media), where the generational consequences of growth burdens manifest slower (Fig 3A). Indeed, the host plays a role in the conjugation efficiency response to changing nutrients. While the MG1655 host maintains an approximate 1:1 correlation between conjugation efficiency in rich TB and poor M9 media, the original pathogen hosts, with the same set of plasmids, do not (Appendix Fig S5A and C). The original pathogen hosts primarily differ in their response to M9 media (Appendix Fig S7). Thus, the plasmid–host relationship is not fully dominated by one side, and hosts can influence how resources are allocated to conjugation.

## Discussion

Up to half of the *E. coli* genome is mobile, as are half of all plasmids, which serve as key mediators of gene flow in microbial communities (Touchon *et al*, 2009; Smillie *et al*, 2010; Brito, 2021). As the reach, influence, and biotechnical applications of plasmids are increasingly appreciated, so too is our poor understanding of the mechanisms determining their prevalence. Vertical and horizontal modes of transfer determine plasmid fitness, and while their interdependence is intuitive, these modes have largely been studied in isolation or with positive selection. Positive selection on an AMR plasmid (i.e., by antibiotics) reduces the cost of the plasmid or makes it beneficial. With increasingly strong selection, the plasmid will persist regardless of transfer rate, obscuring any tradeoff effects.

By studying the interaction of vertical and horizontal transfer across diverse AMR plasmids and environments, we find that plasmid fitness is constrained by a conjugation‐growth tradeoff. Our evidence for the tradeoff is manifold, with integrated genomics, transcriptomics, and phenomics linking conjugation efficiency with *tra* gene expression (Fig 4), *tra* expression with growth effects (Fig 5), and conjugation efficiency with fitness costs (Figs 2 and 3).

Between the two, conjugation efficiency serves as the key differentiating factor and predictor of plasmid fate rather than growth effects. This is due to most AMR plasmids generating little to no growth burden despite their multidrug resistances and novelty to the MG1655 *E. coli* host (Fig 2 and Appendix Figs S3 and S4). In this respect, the host demonstrates a striking tolerance to the increasing *tra* gene expression demands of conjugation efficiencies across multiple orders of magnitude. Such low variation in growth effects suggests that plasmids experience strong natural selection for vertical transfer and evolve in accordance with the tradeoff. Indeed, high conjugation efficiencies appear rare in *E. coli* and selected against the original host pathogens (Appendix Fig S7). These results agree with our simulated and experimental results where conjugation efficiencies near or at the threshold for growth burden are both conducive to high plasmid fitness and prevalent (Fig 3 and Appendix Fig S3).

The emergence of a growth tradeoff at high conjugation efficiencies is similar to what has been observed in plasmid variants generated in laboratory evolution experiments (Turner *et al*, 1998; Dimitriu *et al*, 2021). This apparent alignment between naturally and artificially evolved plasmids supports the possibility of broad threshold correlations between conjugation efficiency and growth effects. As a general source of growth burden, conjugation stands in contrast to more specific plasmid–chromosome sources suggested in the literature (Loftie‐Eaton *et al*, 2017; San Millan *et al*, 2018; Hall *et al*, 2021; Rodríguez‐Beltrán *et al*, 2022), though it does not exclude them. The environment adds additional complexity, shifting the conjugation efficiency threshold for growth burden and how strains balance modes of plasmid transfer. Horizontal transfer generally attracted greater shares of host resources as nutrient levels decreased (Fig 5). This seemingly parasitic behavior exacerbates the growth tradeoff but may also represent an adaptive host response to transient stress or less‐regulated expression (Rodríguez‐Beltrán *et al*, 2021).

Our analysis not only details an evolutionary tradeoff by which the prevalence of plasmids may be manipulated but also a means to leverage it. Most natural plasmids tested here fall within a wide region of host tolerance to conjugative burden. For plasmids in this region, conjugation efficiency may be substantially improved without major fitness costs or inhibited without major fitness benefits. This ability to tune conjugation efficiency to prevent or promote plasmid loss, without growth effects, is desirable in the industry where plasmid loss equates to lost product, and in medicine where plasmids are often the source of antibiotic resistance. Toward these ends, low‐throughput, agar plate‐based quantification of conjugation efficiency has bottlenecked the discovery of plasmid‐targeted treatments (Moralez *et al*, 2021). The massive throughput, multiplexing, and monoculture benefits afforded by RNA‐based quantification therefore have significant potential for the future study and directed manipulation of conjugation, plasmids, and the spread of antibiotic resistance (Brito, 2021; Rodríguez‐Beltrán *et al*, 2021).

## Materials and Methods

### Bacterial strains and plasmids

Data for all strains and plasmids used in this study can be found in Appendix Tables S1 and S2 and BioProjects PRJNA551684 and PRJNA823807. MG1655 *E. coli* strain DA28102 has the following genotype: F‐λ‐*ilvG*‐*rfb‐50 rpb‐1 galK::cat‐*J23101‐mTagBFP2. Similarly, the genotype of MG1655 *E. coli* strain fAYC002 is as follows: *PRO ΔcsgA ompR234*.

### Growth media

M9, M9 with 2 mg/ml casamino acids (M9CA), Luria‐Bertani (LB, Miller), and Terrific Broth (TB) were used across all experiments. Both M9 and M9CA were supplemented with 0.1 mg/ml thiamine, 2 mM MgSO_4_, 0.1 mM CaCl_2_, and 0.4% w/v glucose. Carbenicillin (Carb, 100 μg/ml) and chloramphenicol (Cm, 50 μg/ml) were added for plasmid and MG1655 *E. coli* strain DA28102 selection, respectively. Kanamycin (Kan, 50 μg/ml) was added to select MG1655 *E. coli* strain fAYC002. Unless otherwise stated, all liquid cultures were started from colonies on LB agar with selection.

### Growth rate measurements

Standard competition assay measurements of host fitness cost are confounded by conjugation, which converts plasmid‐free cells to plasmid‐carrying cells (San Millan, 2018). Instead, we used host growth rates with and without plasmid to broadly measure growth effects (Vogwill & MacLean, 2015; Alonso‐del Valle *et al*, 2021; Prensky *et al*, 2021; Rajer & Sandegren, 2022). For consistency in starting cell density and state, strains were first grown in 1 ml LB media, shaking for 18 h at 37°C. Cultures were then diluted 100‐fold into M9, M9CA, or TB media, covered with 50 μl mineral oil, and grown with shaking at 37°C in a plate reader for 24 h. OD_600_ measurements were taken approximately every 10 min. The subsequent growth data were blank subtracted, smoothed with an equally weighted moving average filter, and log transformed before calculating the maximum growth rate (Appendix Fig S1). Growth rate measurements were normalized to a standard DA28102 culture to account for any inconsistencies across equipment.

### Conjugation efficiency measurements

MG1655 strains DA28102 and fAYC002 served as plasmid donor and recipient, respectively, in all experiments except those in Appendix Fig S7. Conditions during conjugation were tailored to limit confounding growth dynamics; thus, media were varied during the initial culture. Donor and recipient strains were first grown in 3 ml M9/M9CA/TB media, shaking for 18 h at 37°C. Donors were then diluted 2.5/5/10‐fold in kind for M9/M9CA/TB media, respectively, and grown for 2 h at 37°C without shaking. This is to allow pili formation and conjugation of IncF plasmids during the exponential phase. Following regrowth, donors and recipients were pelleted at 2,000 rcf for 10 min at room temperature. We resuspended donor pellets in M9CA for conjugation, concentrating them by 10/5/4‐fold if they were previously grown in M9/M9CA/TB media. Recipient pellets were also resuspended in M9CA and concentrated by 4/2/1‐fold in the same way. These concentrations aimed to balance donor and recipient densities in an approximate 1:1 ratio, adjusted accordingly based on OD_600_. Now in M9CA for growth‐controlled conjugation, donors and recipients were mixed 1:1 for 1 h at room temperature. Donor and recipient cultures were plated on LB agar with selection to measure parent densities, followed by transconjugants at the end of the 1 h conjugation period. Cell mixtures were vortexed at the end of the 1 h conjugation period to disrupt further conjugation during transconjugant plating. Conjugation efficiency was calculated as η $=\frac{T}{\mathrm{DR}\Delta t}$, with transconjugant (T), donor (D), and recipient (R) cell densities over time (t).

### Modeling plasmid dynamics

Our plasmid fitness model has two ordinary differential equations that incorporate growth and conjugation dynamics of the plasmid‐free (*s*_0_) and plasmid‐carrying (*s*_1_) cell populations. Parameters include *μ* (hr^−1^), specific growth rates for each population, normalized *η* (h^−1^), plasmid conjugation efficiency, κ (h^−1^), plasmid loss rate, and *D* (h^−1^), the dilution rate of the system.

$$\frac{ds_{0}}{\mathrm{dt}}=\mu_{0}s_{0}\left(1-s_{0}-s_{1}\right)-\eta s_{0}s_{1}+\kappa s_{1}-Ds_{0}$$

$$\frac{ds_{1}}{\mathrm{dt}}=\mu_{1}s_{1}\left(1-s_{0}-s_{1}\right)+\eta s_{0}s_{1}-\kappa s_{1}-Ds_{1}$$

We make several simplifying assumptions. First, that the plasmid loss due to segregation error is negligible relative to other factors. Second, that the conjugation efficiency and growth effects do not change over time. Growth rates were defined as maximums and conjugation efficiencies were measured during the exponential phase; therefore, both may overestimate the average along the entire time course. Finally, dilution is applied continuously instead of stepwise as done in long‐term experiments.

Parameter values and initial population sizes were matched to experimental protocols where possible. Carrying capacity was set to 1 for simplicity, starting cell densities were diluted 1,000‐fold per experimental protocol, conjugation efficiencies were varied across the measured range, plasmid‐free growth rates can be found in Appendix Table S2, and growth effects were inferred from piecewise fits in Appendix Fig S2. Dilution rate and simulation time were set to 0.01 and 10,000, respectively.

### Plasmid fitness measurements

DA28102 strains with and without plasmids were cultured in 3 ml LB media with appropriate selection and shaking at 37°C for 18 h. Strains 41T, 92T, 94T, 2350T, 2629T, 3204T, 4219T, 4563T, 4592T, and 5696T were selected for simple plasmid compositions (i.e., one transferrable, beta‐lactamase carrying plasmid), a wide range of conjugation efficiencies, and diverse Inc groups. Plasmid‐free and plasmid‐carrying strains were then serially diluted 1,000‐fold and mixed 1:1 in a deep well plate (DWP) with 1 ml TB and M9 media wells. The plate was covered with two gas‐permeable membranes to limit evaporation and incubated at 37°C with shaking at 700 rpm for 24 h. The plate was passaged for 3 days with 100,000‐fold dilutions for longer exponential phases, evolutionary bottlenecking, and more generations per day. Starting and final cell densities were determined through agar plating with differential selection for plasmid‐carrying and plasmid‐free cells.

### Genome sequencing, assembly, and analysis

We sequenced the remaining 17 clinical *E. coli* isolates with conjugative phenotypes screened in Bethke *et al* (2020) and our MG1655 recipient DA28102 for a complete genomic profile of the plasmids and strains tested here. Genomic DNA was extracted with the QIAGEN MagAttract high molecular weight DNA kit (Cat ID: 67563) from cultures grown for 16 h until an approximate density of 10^9^ cells/ml. DNA libraries were prepared according to PacBio's recommendations and sequenced using the PacBio Sequel system.

Raw reads were assembled and polished using both HGAP4 (SMRTLink v6.0.0.47841) and Flye (v2.4.2). Default parameters were used with the exception of the following in HGAP4: Aggressive:TRUE Falcon configuration over‐ride: pa_DBsplit_option = −x500 ‐s200; ovlp_DBsplit_option = ‐x500 ‐s200; falcon_sense_option = ‐‐output_multi ‐‐min_idt 0.70 ‐‐min_cov 2 ‐‐max_n_read 200 ‐‐n_core 6; overlap_filtering_setting = ‐‐max_diff 100 ‐‐max_cov 100 ‐‐min_cov 2 ‐‐bestn 10 ‐‐n_core 24; pa_HPCdaligner_option = ‐v ‐B4 ‐t16 ‐e.70 ‐l1000 ‐s1000; ovlp_HPCdaligner_option = ‐v ‐B4 ‐t32 ‐h60 ‐e.96 ‐l500 ‐s1000. Assemblies were evaluated with QUAST (v5.0.2) and BUSCO (v3.0.2) with the final assembly chosen based on N50 and the number of BUSCOs identified. Genome annotations were performed using RAST and Prokka v1.13 (Aziz *et al*, 2008; Seemann, 2014). Inc, MOB, MLST, and resistance typing were performed as described previously (Zankari *et al*, 2012; McArthur *et al*, 2013; Orlek *et al*, 2017; Bethke *et al*, 2020).

### RNA sequencing and analysis

Strains ESBL41T, ESBL92T, GN02560T, GN05243T, and pCU1T were prepared in the same manner as for conjugation efficiency measurements. At the conjugation phase, cells were instead frozen and submitted to Genewiz for RNA extraction and sequencing. All strain samples underwent rRNA depletion and QC for RIN scores > 6. Whole transcriptome, 150 base paired‐end sequencing was performed using the Illumina HiSeq platform with > 25 M reads per sample. Per FastQC, the mean quality score was 38.26 with 91.94% bases > = 30. Reads were trimmed using Trimmomatic v0.36 and mapped to MG1655 and pathogen reference genomes using Bowtie2 v2.2.6. Unique gene hit counts were calculated using Subread package v1.5.2 and analyzed via transcripts per million (TPM) and DESeq2 (Love *et al*, 2014).

## Author contributions

**Jonathan H Bethke:** Conceptualization; data curation; formal analysis; funding acquisition; validation; investigation; visualization; methodology; writing – original draft; project administration; writing – review and editing. **Helena R Ma:** Formal analysis; investigation; writing – original draft. **Ryan Tsoi:** Formal analysis; investigation. **Li Cheng:** Formal analysis; investigation; methodology. **Minfeng Xiao:** Supervision; funding acquisition; project administration. **Lingchong You:** Conceptualization; supervision; funding acquisition; visualization; writing – original draft; project administration; writing – review and editing.

## Disclosure and competing interests statement

The authors declare that they have no conflict of interest. LY is an editorial advisory board member. This has no bearing on the editorial consideration of this article for publication.

## Supporting information



Appendix S1

## Data Availability

All data needed to evaluate the conclusions in the paper are in the paper and/or the Appendix. Genomic sequence data are available from GenBank with BioProject accession numbers PRJNA551684 (https://www.ncbi.nlm.nih.gov/bioproject/PRJNA551684) and PRJNA823807 (https://www.ncbi.nlm.nih.gov/bioproject/PRJNA823807). RNAseq data are available from GEO with accession number GSE219270 (http://www.ncbi.nlm.nih.gov/geo/query/acc.cgi?acc=GSE219270). Additional data available at https://github.com/youlab/PlasmidTradeoff_Bethke.
