# Supplementary material for: Vertical and horizontal gene transfer tradeoffs direct plasmid fitness
Source: Mol Syst Biol. 2022 Dec 27;19(2):e11300. doi: 10.15252/msb.202211300 (PMC9912019; doi:10.15252/msb.202211300)
Supplement: Supplementary file 1 — Appendix S1 [file MSB-19-e11300-s002.pdf]

APPENDIX

Table of Contents:

Appendix Figures S1 to S8 pgs. 2-9
Appendix Tables S1 to S2 pgs. 10-14

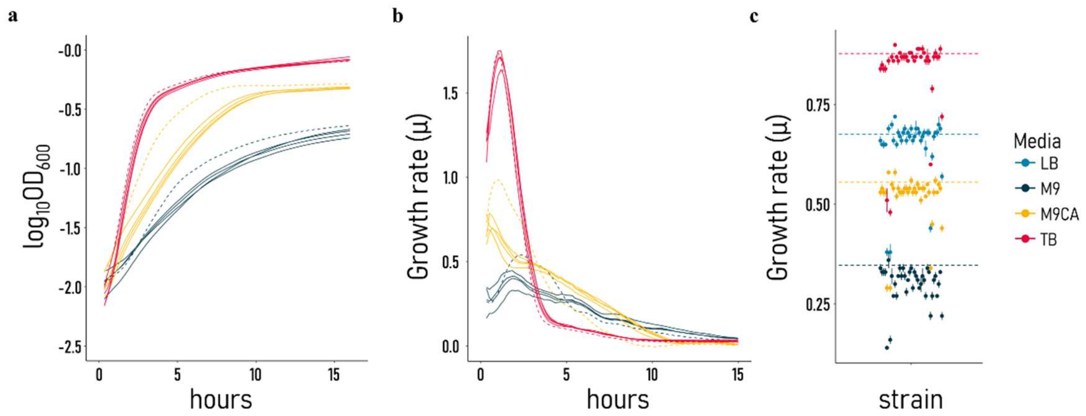

Appendix Figure S1. Measuring plasmid growth effects. (A) We obtained OD_{600} growth curves in plate readers across varied media (red TB, yellow M9CA, dark blue M9) at 37°C with shaking. Plasmid-carrying MG1655 *E. coli* (solid lines) were grown alongside plasmid-free controls (dashed lines) in at least triplicate for comparison. Raw growth curves were blank-subtracted, smoothed with a weighted moving average filter, and log transformed. (B) Growth rates (μ, hr^{-1}) were determined across growth curves to find the maximum growth rate for each condition and replicate. (C) Average maximum growth rates were normalized by the appropriate plasmid-free control for that day and equipment to represent the plasmid growth effect (ω) = $1 - (\mu_{p+}/\mu_{p-})$. Each point represents the average of at least triplicate measurements per transconjugant strain ± SD.

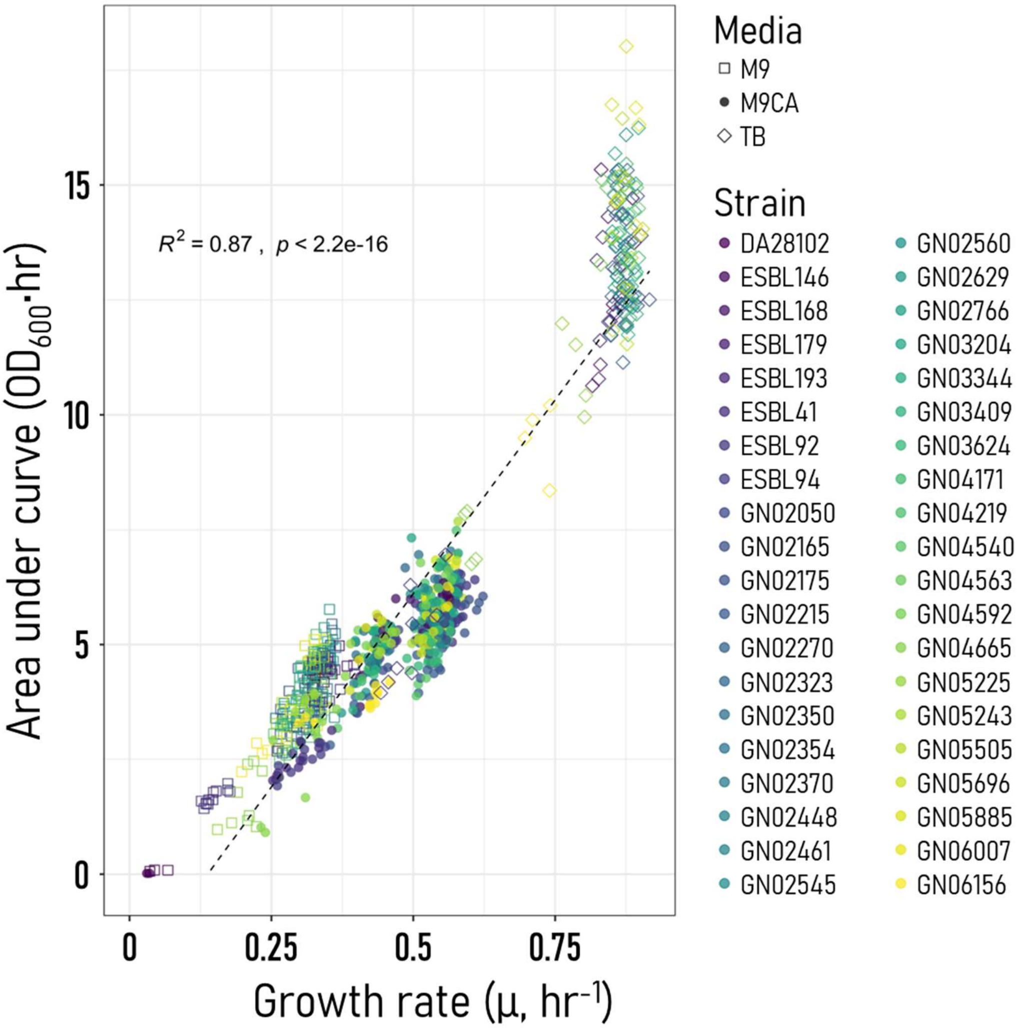

Appendix Figure S2. Correlation between area under curve and specific growth rate. Specific growth rates (μ, hr^{-1}) and areas under growth curves (OD_{600}•hr) are shown from all growth curves collected (n = 881). Growth data include biological replicates in M9, M9CA, and TB media with and without 100 Carb (100 μg/mL) and Cm (50 μg/mL) antibiotics. Growth rates were calculated as in Appendix Figure 1. Areas under growth curves were calculated from blank subtracted and weighted moving average smoothed data.

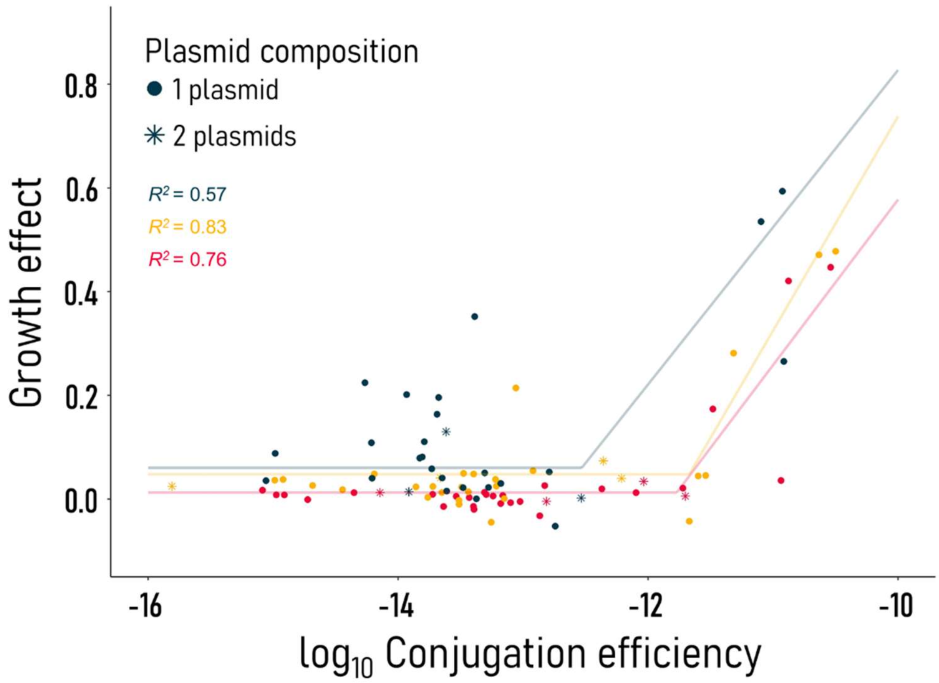

Appendix Figure S3. Tradeoff between conjugation efficiency and plasmid growth effect across growth media. Piecewise relationship between conjugation efficiency (η, $(\text{cells/mL})^{-1}\text{hr}^{-1}$) and growth effect (ω) of MG1655 *E. coli* transconjugants carrying diverse AMR plasmids, measured in M9 (blue), M9CA (yellow), and TB (red) growth media. Points are shaped by plasmid composition and represent the average of at least triplicate measurements.

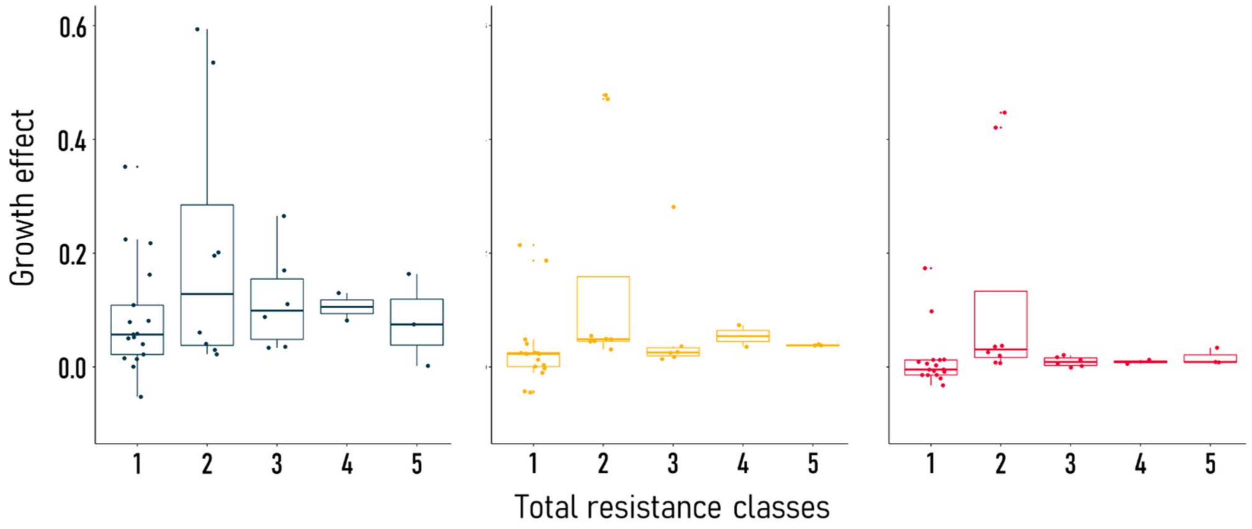

Appendix Figure S4. Total unique classes of antibiotic resistance do not correlate with plasmid growth effect. A one-way ANOVA revealed no significant difference in plasmid growth effect between total antibiotic resistance classes as determined by ResFinder and grouped by growth media ($F(8, 451) = 0.366$, $P = 0.938$). M9 (blue), M9CA (yellow), and TB (red) growth media are displayed.

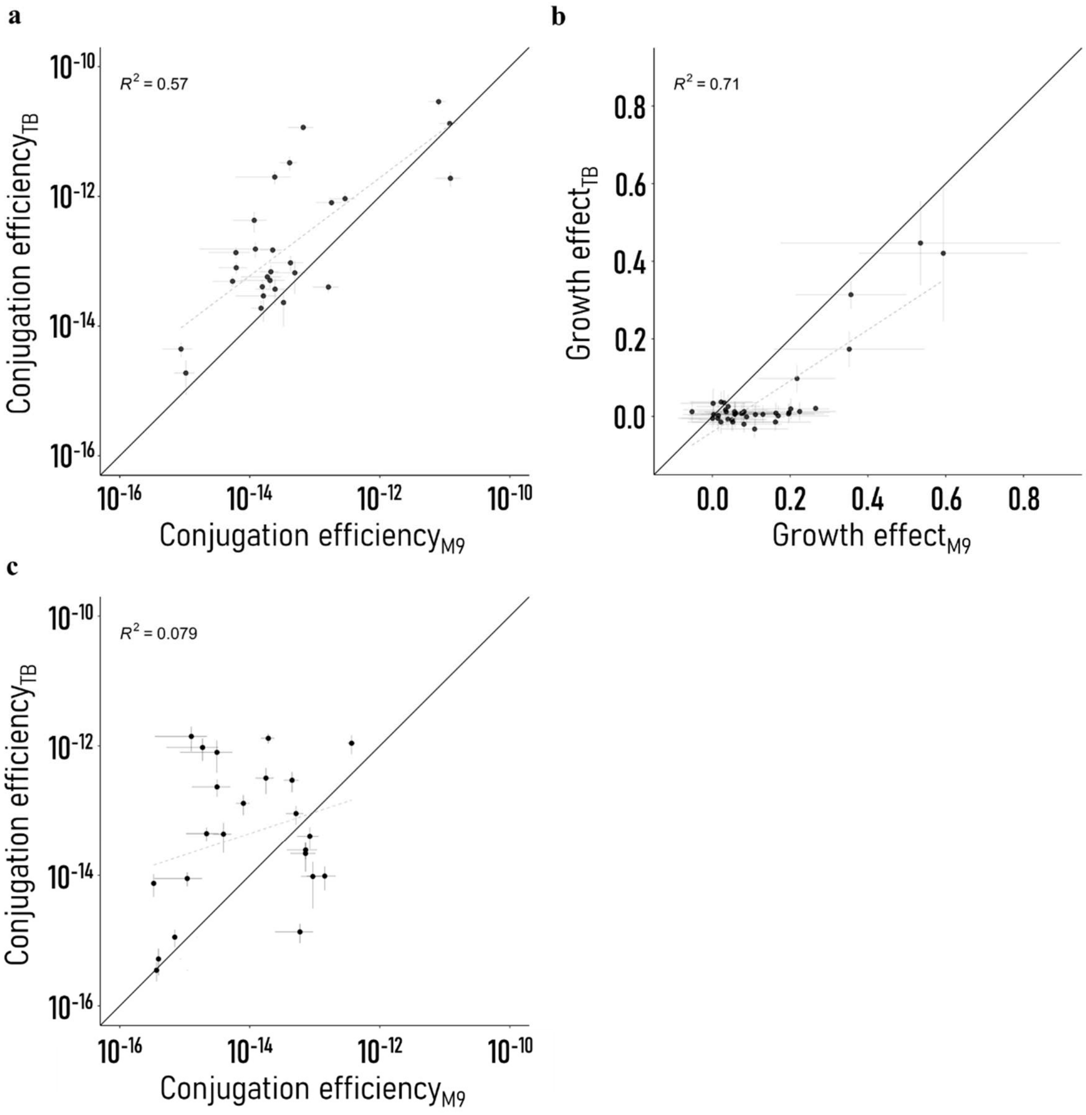

Appendix Figure S5. Nutrient effect on conjugation efficiency and plasmid growth effect. (A) Per strain MG1655 *E. coli* transconjugant conjugation efficiencies ((cells/mL)$^{-1}$hr^{-1}) in nutrient rich TB media compared against those in nutrient poor M9 media. (B) Per strain MG1655 *E. coli* transconjugant growth effects in TB versus M9 media. (C) Per strain *E. coli* pathogen conjugation efficiencies in TB versus M9 media. Points are averages of at least triplicate measurements +/- SD. Solid lines indicate 1:1 parity between conditions.

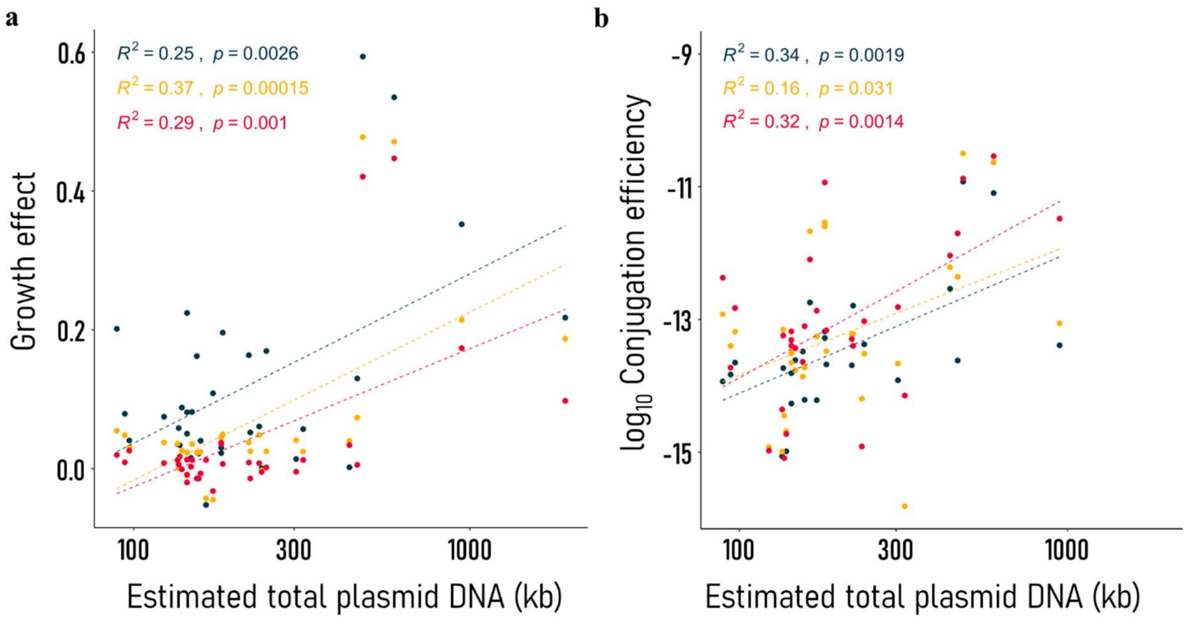

Appendix Figure S6. Correlations with estimated total plasmid DNA. Total plasmid DNA was estimated from sequence length and average sequence coverage relative to the bacterial chromosome. Cultures for sequencing were grown in LB media. (A) The relationship between estimated total plasmid DNA and plasmid growth effect. Average growth effects (n = 3-5 biological replicates) are shown for M9 (blue), M9CA (yellow), and TB (red) media. (B) The relationship between estimated total plasmid DNA and conjugation efficiency. Average conjugation efficiencies (n = 3-6 technical replicates, 1-2 biological replicates) are shown for each media condition.

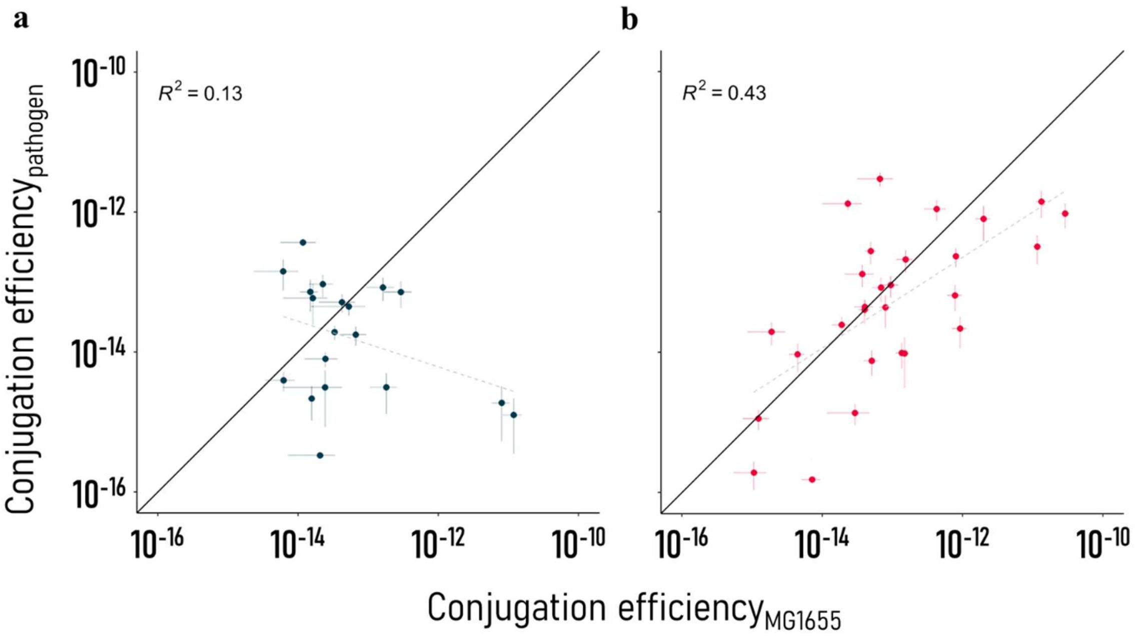

Appendix Figure S7. Host effects on vertical and horizontal gene transfer. Conjugation efficiency ((cells/mL)$^{-1}$hr^{-1}) comparison between the original pathogenic and MG1655 *E. coli* in (A) nutrient poor M9 and (B) rich TB media. All points represent the average of at least triplicate measurements ± SD. Solid lines indicate 1:1 parity between conditions. Dashed lines are trendlines between pathogen and MG1655 conjugation efficiencies.

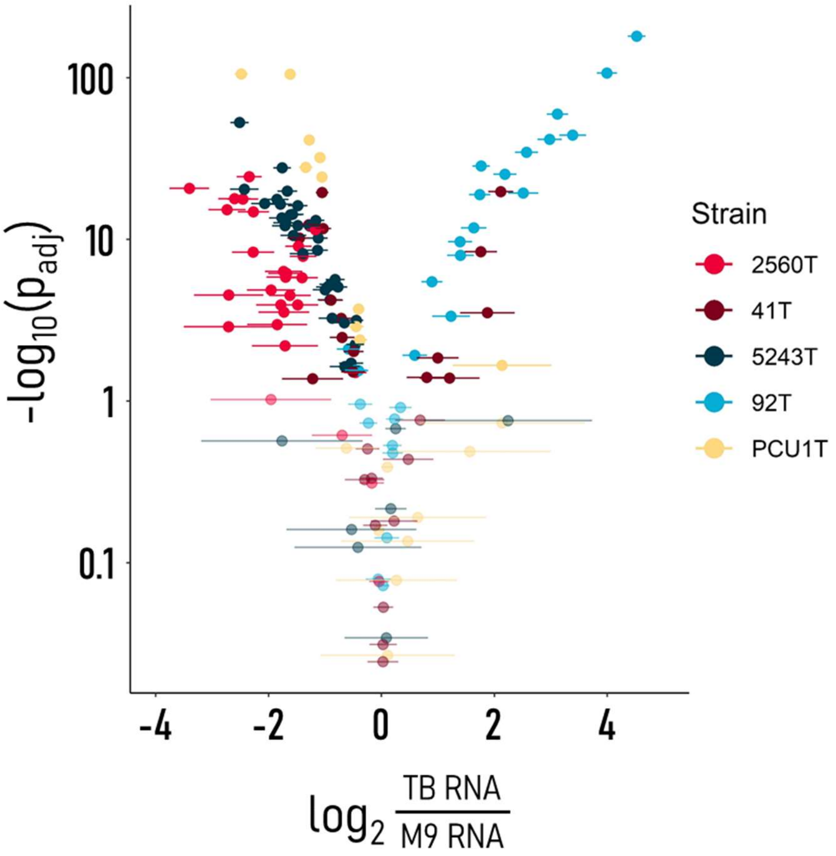

Appendix Figure S8. **Expression of genes associated with HGT by strain.** Volcano plot from Figure 5 colored by strain.

Strain	Sequence Type (ST)	fimH	Plasmid Size (kb)	MOB Relaxase	Incompatibility (Inc) Group	Antibiotic Resistance	Transconj. Presence	Est. Copy #	Reference
DA28102	10	H27	-	-	-	P	-	-	Gullberg et al.
fAYC002	-	-	-	-	-	A	-	-	Chen et al.
pCU1	-	-	-	-	IncN	A, B	+	-	Sysoeva et al.
ESBL41	131	H30	121	MOBF	IncFIB/IncFIA/IncFII	P	-	-	Bethke et al.
			120	MOBF	IncN/IncFII	B, P	+	4	
ESBL92	3057	H445	82	MOBP	IncFII/IncI1	B	+	2	Bethke et al.
ESBL94	131	H30	121	MOBF	IncFIB/IncFIA/IncFII	P	-	-	Bethke et al.
			119	MOBF	IncN/IncFII	B, P	+	5	
ESBL146	131	H30	100	MOBF	IncFIB/IncFIA/IncFII	A, B, M, S, T	+	3	Bethke et al.
			97	-	IncY	-	-	-	
			69	MOBF	IncFII	-	-	-	
			46	MOBF	IncN	-	+	3	
ESBL168	405	H27	131	MOBF	IncFIB/IncFII/Col156	P	-	-	Bethke et al.
			109	-	IncFIB	-	-	-	
			97	MOBP	IncB/O/K/Z	B, S	+	1	
			92	MOBF	IncQ1/IncFII	S, T	-	-	
			60	-	IncI2	-	-	-	
			57	MOBF	IncN2	-	-	-	
			34	MOBF	IncN2	-	-	-	
ESBL179	131	H30	122	MOBF	IncFIA/IncFII	A, S, T	-	-	Bethke et al.
			91	MOBP	IncI1	A, B	+	2	
			72	MOBF	IncFII	B	-	-	
ESBL193	131	H30	122	MOBF	IncFIA/IncFII	A, S, T	-	-	Bethke et al.
			91	MOBP	IncI1	A, B	+	2	
GN02050	144	H54	121	MOBF	IncFIB/IncFII/Col156_1	-	-	-	This study
			68	MOBF	IncFIC(FII)[a]	B	+	2	
			16	MOBF	IncFIC(FII)[a]	-	-	-	
			12	MOBP	ColRNAI_1[a]	-	-	-	
			11	MOBQ	Col156_1	-	-	-	
			7	MOBQ	Col156_1	-	-	-	
			6	cat	ColRNAI_1[a]	-	-	-	
GN02165	95	H107	176	MOBF	IncFIB/IncFIC(FII)/IncFIA	-	-	-	This study
			72	MOBF	IncFIC(FII)[a]	B	+	2	
			44	MOBF	IncN	-	-	-	
			35	MOBQ	IncX5_2[a]	-	-	-	
			21	-	IncN	-	-	-	
GN02175	144	H54	145	MOBF	IncFIB/IncFII/Col156	P	-	-	Bethke et al.
			86	MOBP	IncB/O/K/Z	B	+	2	
			34	MOBQ	IncX1	-	-	-	

			29	-	IncFII	-	-	-	
			10	-	-	B	-	-	
GN02270	69	H27	165	MOBF	IncFIB/IncFII	B	+	1	This study
			7	MOBP	ColRNAI_1[a]	-	+	22	
			5	MOBQ	Col156_1	-	-	-	
GN02323	73	H30	114	MOBF	IncFIB/IncFII/Col156_1	P	-	-	This study
			74	MOBF	IncFIC(FII)[a]	B	+	2	
			6	-	ColRNAI_1[a]	-	+	26	
GN02350	12	H27	118	MOBF	IncFIB/IncFII/Col156_1	B, S	+	2	This study
GN02448	131	H22	124	MOBF	IncFIB/IncFIA/IncFII	B, P, S	+	2	Bethke et al.
			98	-	p0111	-	-	-	
			54	MOBF	IncN[a]	-	-	-	
GN02461	68	H382	149	MOBF	IncFIB/IncFII/IncQ1	A, B, S, T	+	1	This study
			78		IncFII(pECLA)[a]/IncI1_1_Alpha	B	-	-	
			16	MOBQ	Col156_1	-	-	-	
			7	MOBP	ColRNAI_1[a]	-	-	-	
GN02545	131	H22	79	MOBF	IncFII	B	+	2	Bethke et al.
GN02560	131	H30	136	MOBF	IncFIB/IncFIA/IncFII/Col156_1	B	-	-	
			80	MOBF	IncFII	B	+	3	
			14	MOBP,MOBQ	Col156_1	-	-	-	
			7	MOBQ	Col156_1	-	-	-	
			6	MOBQ	Col156_1	-	-	-	
			5	-	Col(MG828)_1/ColpVC_1	-	-	-	
GN02629	131	H30	110	MOBF	IncFIA/IncFII	A, B, M, S, T	+	2	Bethke et al.
			109	-	IncFIB	-	-	-	
GN02766	73	H9	137	MOBF	IncFIB/IncFII/Col156	A, B, S	+	1	Bethke et al.
GN03204	131	H41	123	MOBF	IncFIB/IncFII/Col156_1	A, B, M, S, T	+	1	This study
GN03344	131	H30	131	MOBF	IncFIB/IncFIA/IncFII/Col156_1	B	-	-	
			72	MOBF	IncFIC(FII)[a]	B	+	2	
			19	MOBQ	Col156_1	-	-	-	
			13	MOBP	ColRNAI_1[a]	-	-	-	
			6	MOBP	Col440II_1[a]/ColRNAI_1	-	-	-	
GN03409	95	H18	77	MOBF	IncFII	B	+	2	Bethke et al.
			76	MOBF	IncFIB/IncFII	-	-	-	
GN03624	58	H27	191	MOBF	IncFIB/IncFIC(FII)	-	-	-	Bethke et al.
			119	-	IncFII	A, S, T	+	2	
			112	MOBP	IncI1	A, B, S	+	2	
GN04171	131	H30	74	MOBF	IncFIC(FII)[a]	B	+	3	This study
			68		IncFIB/IncFIA/IncFII	B	-	-	
			43	MOBF	IncN_1	-	-	-	
			6	-	ColRNAI_1[a]	-	-	-	
GN04219	69	H27	139	MOBF	IncFIB/IncQ1/IncFII/Col156	B, S, T	+	1	This study

GN04540	963	H26	94	MOBP	IncFII(pECLA)/IncI1	B	-	-	Bethke et al.
			89	MOBF	IncFII	A, B	+	1	
GN04563	131	H30	74	MOBF	IncFIC(FII)	B	+	2	Bethke et al.
			44	-	IncFIB/IncFIA	T	-	-	
GN04592	69	H27	135	MOBF	IncFIB/IncQ1/IncFII/Col156	B, S, T	+	1	This study
GN05225	69	H27	113	MOBF	IncFIB/IncFIA/IncFII	B	+	17	This study
GN05243	131	H30	127	-	IncFIA/IncFII	A, M, S, T	-	-	This study
			94	MOBP	IncB/O/K/Z	B	+	1	
			62	MOBF	IncN_1	T	-	-	
			38	MOBP	IncX4_2	-	-	-	
			22	-	-	T	-	-	
GN05505	69	H27	125	MOBF	IncFIB/IncFII/Col156	-	-	-	This study
			72	MOBF	IncFIC(FII)[a]	B	+	2	
			5	MOBQ	Col156_1	-	-	-	
GN05696	95	H27	92	MOBF	IncN/IncFII	B, S	+	2	Bethke et al.
			15	-	Col	-	-	-	
GN05885	131	-	120	MOBP	IncB/O/K/Z	B, T, S	+	-	Bethke et al.
			79		IncFII	B, P	-	-	
GN06007	69	H27	93	MOBF	IncFIB/IncFII	A, B, S, T	-	-	Bethke et al.
			78	MOBF	IncFII(pCoo)	B	+	2	
			32	MOBF	IncN	-	-	-	
GN06156	69	H47	90	MOBP	IncI1_1_Alpha	-	-	-	This study
			63	MOBF	IncFII(pCoo)_1	B	+	15	

A, aminoglycoside; B, beta-lactam; M, macrolide; P, phenicol; S, sulfonamide; T, tetracycline

<90% sequence identity[a]

Appendix Table S1. **Strains and plasmids used in this study.** Pathogen donor strains were sequenced and are listed here with plasmid-free recipient strains DA28102 and fAYC002. The transconjugant DA28102 strains carrying AMR plasmids are indicated by a #T nomenclature throughout the text. Antibiotics abbreviations are as follows: A, aminoglycoside; B, beta-lactam; M, macrolide; P, phenicol; S, sulfonamide; T, tetracycline. < 90% sequence identity [a].

	M9			M9CA			TB		
**Strain**	**μ**	**ω**	**η**	**μ**	**ω**	**η**	**μ**	**ω**	**η**
DA28102	0.342	NA	NA	0.554	NA	NA	0.874	NA	NA
ESBL41T	0.139	0.594	1.19E-11	0.289	0.478	3.17E-11	0.506	0.421	1.33E-11
ESBL92T	0.360	-0.052	1.81E-13	0.577	-0.043	2.14E-12	0.863	0.012	8.02E-13
ESBL94T	0.159	0.535	8.03E-12	0.293	0.471	2.33E-11	0.483	0.447	2.89E-11
ESBL146T	0.341	0.002	2.92E-13	0.532	0.040	6.14E-13	0.845	0.034	9.23E-13
ESBL168T	0.328	0.041	2.25E-14	0.537	0.031	6.56E-14	0.852	0.026	1.49E-13
ESBL179T	0.334	0.022	5.29E-14	0.529	0.045	2.52E-12	0.842	0.037	-
ESBL193T	0.332	0.030	6.64E-14	0.529	0.045	2.89E-12	0.843	0.036	1.16E-11
GN02050T	0.322	0.058	1.86E-14	0.553	0.001	7.07E-14	0.869	0.006	5.75E-14
GN02165T	0.265	0.224	5.43E-15	0.541	0.023	3.22E-14	0.863	0.013	4.91E-14
GN02175T	0.305	0.108	6.12E-15	0.579	-0.045	5.56E-14	0.902	-0.032	1.36E-13
GN02215T	0.275	0.197	-	0.520	0.060	-	0.865	0.010	-
GN02270T	0.322	0.057	-	0.540	0.025	1.55E-16	0.863	0.012	7.17E-15
GN02323T	0.337	0.014	1.22E-14	0.531	0.041	2.18E-14	0.878	-0.005	1.54E-13
GN02350T	0.321	0.061	-	0.527	0.048	6.44E-15	0.867	0.008	1.23E-15
GN02354T	0.341	0.003	-	0.534	0.036	-	0.870	0.005	-
GN02370T	0.322	0.058	-	0.541	0.023	-	0.869	0.006	7.79E-13
GN02448T	0.284	0.170	-	0.540	0.025	-	0.873	0.002	-
GN02461T	0.314	0.082	-	0.534	0.035	-	0.863	0.013	-
GN02545T	0.328	0.040	6.19E-15	0.540	0.024	1.90E-14	0.880	-0.007	7.95E-14
GN02560T	0.342	0.000	4.23E-14	0.555	-0.002	3.07E-14	0.878	-0.005	9.46E-14
GN02629T	0.286	0.163	2.05E-14	0.533	0.038	6.01E-14	0.867	0.009	5.07E-14
GN02766T	0.330	0.034	-	0.544	0.018	3.59E-15	0.859	0.017	8.21E-16
GN03204T	0.316	0.075	-	0.533	0.038	1.20E-15	0.867	0.008	1.06E-15
GN03344T	0.314	0.081	1.56E-14	0.547	0.013	2.24E-14	0.892	-0.020	4.05E-14
GN03409T	0.287	0.162	-	0.541	0.023	-	0.887	-0.014	-
GN03624T	0.298	0.130	2.42E-14	0.513	0.073	4.39E-13	0.869	0.006	1.99E-12
GN04171T	0.324	0.052	1.62E-13	0.540	0.025	6.12E-14	0.887	-0.014	4.01E-14
GN04219T	0.312	0.088	1.04E-15	0.539	0.026	2.07E-15	0.875	-0.001	1.89E-15
GN04540T	0.273	0.201	1.17E-14	0.524	0.055	1.20E-13	0.857	0.020	4.27E-13
GN04563T	0.337	0.015	2.45E-14	0.552	0.003	1.73E-14	0.872	0.003	3.71E-14
GN04592T	0.330	0.035	8.76E-16	0.534	0.036	1.03E-15	0.863	0.012	4.44E-15
GN04665T	0.220	0.356	-	0.343	0.380	-	0.600	0.314	-
GN05225T	0.268	0.218	-	0.450	0.187	-	0.789	0.098	-
GN05243T	0.315	0.079	1.49E-14	0.527	0.048	4.02E-14	0.866	0.009	1.89E-14
GN05505T	0.325	0.050	4.93E-14	0.559	-0.010	3.08E-14	0.882	-0.009	6.62E-14
GN05696T	0.275	0.196	2.11E-14	0.526	0.049	3.34E-14	0.868	0.007	6.88E-14
GN05885T	0.304	0.111	1.62E-14	0.546	0.014	3.65E-14	0.870	0.005	2.92E-14
GN06007T	0.334	0.022	3.31E-14	0.541	0.023	1.39E-14	0.887	-0.014	2.31E-14
GN06156T	0.222	0.352	4.10E-14	0.435	0.214	8.76E-14	0.722	0.174	3.30E-12
pCU1T	0.251	0.265	1.22E-11	0.398	0.281	4.83E-12	0.856	0.021	1.90E-12

Appendix Table S2. **Strain specific growth rates (μ, hr^{-1}), plasmid growth effects (ω), and conjugation efficiencies (η, $(cells/mL)^{-1}hr^{-1}$).** DA28102 transconjugant strains indicated by #T, with plasmid growth effects relative to plasmid-free DA28102 in each media. Growth data are averages of 3-5 biological replicates. Conjugation efficiency data are averages of 3-6 technical replicates and 1-2 biological replicates.
