# Supplementary material for: Vertical and horizontal gene transfer tradeoffs direct plasmid fitness
Source: Mol Syst Biol. 2022 Dec 27;19(2):e11300. doi: 10.15252/msb.202211300 (PMC9912019; doi:10.15252/msb.202211300)
Supplement: Supplementary file 2 [file MSB-19-e11300-s001.pdf]

molecular systems biology

Vertical and horizontal gene transfer tradeoffs direct plasmid fitness

Jonathan Bethke, Helena Ma, Ryan Tsoi, Li Cheng, Minfeng Xiao, and Lingchong You
DOI: 10.15252/msb.202211300

Corresponding author(s): Lingchong You (you@duke.edu)

Review Timeline:

Submission Date:	15th Aug 22
Editorial Decision:	9th Sep 22
Revision Received:	8th Nov 22
Editorial Decision:	2nd Dec 22
Revision Received:	11th Dec 22
Accepted:	12th Dec 22

Editor: Maria Polychronidou

Transaction Report:

(Note: With the exception of the correction of typographical or spelling errors that could be a source of ambiguity, letters and reports are not edited. Depending on transfer agreements, referee reports obtained elsewhere may or may not be included in this compilation. Referee reports are anonymous unless the Referee chooses to sign their reports.)

1st Editorial Decision 9th Sep 2022

Thank you again for submitting your work to Molecular Systems Biology. We have now heard back from the three reviewers who agreed to evaluate your study. As you will see below, the reviewers think that the study is a relevant contribution to the field.

However, they raise a series of concerns, which we would ask you to address in a revision.

I think that the reviewers' comments are rather clear and straightforward to address. I therefore see no need to repeat any of the comments listed below.

All issues raised by the referees would need to be satisfactorily addressed. Please let me know in case you would like to discuss in further detail any of the issues raised, I would be happy to schedule a call.

On a more editorial level, we would ask you to address the following points:

Reviewer #1:

In this study, Bethke et al. investigate how the trade-off between vertical and horizontal modes of plasmid transfer impact plasmid fitness. This is quite an interesting topic which has been discussed a lot but had not been property assessed until now. This work demonstrates that this trade-off is conserved for multiple wild type plasmids, but that it is only evident from a given threshold of conjugation efficiency (a quite high conjugation rate, actually). This result has important implications for understanding how plasmids are able to survive in bacterial populations. Moreover, the authors show that the expression levels of conjugation genes do in fact correlate with conjugation efficiency, and ultimately with plasmid fitness effects (at least once passed the conjugation threshold), and propose that expression levels could therefore be used to predict these phenotypes. In addition, the authors also used mathematical models that generally agree with their experimental results, in order to generalize their findings. In general, I think that this is an interesting work and would represent an important contribution to the field. I present some minor comments below:

Some of the key experiments of this study are obviously the conjugation assays. If I understood correctly, the authors conjugated the AMR plasmids from the wild-type enterobacteria to MG1655, and then they calculated the transfer efficiencies between MG1655. I was not able to find the details of the donor and recipient strains (how did the authors differentiate between donor and recipient MG1655, for example) in the methods section. Maybe the authors could provide a little more detail about it?

This is just a suggestion: In our experience sometimes the area under the growth curve provide a better proxy for fitness than the maximum growth rate. Have you tried running your analyses using the AUC instead of Vmax?
I think it the term "pathogen plasmid" may be a bit less accurate than maybe "AMR plasmid" or "resistance plasmid", since the plasmid is selected for the AMR mechanism.

Supp figure 3. Plasmid size could be multiplied by plasmid copy number (inferred from the plasmid/chromosome coverage data from genome sequences) to check if the total extra DNA content correlates with plasmid growth effect, as a possible additional test.

Define the dashed line in Supp Fig. 6

Reviewer #2:

In this study, the authors measure conjugation cost and study growth-conjugation trade-offs across 40 plasmids from clinical E. coli. They present evidence that conjugation becomes costly at high rates; and that most plasmids transfer at rates below this level. They also show that the cost of conjugation on bacterial growth is stronger in poor medium, and link this cost to transfer gene expression.
The idea of a trade-off is not novel, but this study provides a well-executed and interesting, in-depth characterisation of this trade-off across clinical plasmids, with new results on the molecular causes for the cost of conjugation. I have no major criticisms, but some divergences in interpretation with the authors, and a few minor comments to improve clarity.

- first, I am not convinced by the use of the word 'threshold' to describe the increase in cost only at high conjugation rates. Does the cost still exhibit such visible threshold if conjugation rates are plotted in linear scale? I don't find it surprising, or striking, that such threshold appears to be present when conjugation rates are on a log scale while the effect on growth is effectively a linear measure. I agree with the authors that conjugation rates are usually presented with a log scale, because they can be very small indeed, but not detecting any significant difference between the costs conferred by a 10000th and a 100000th of the maximal transfer efficiency (which is around 10-9 to 10-8) across noisy biological systems is only to be expected.

- I also don't agree with the way the authors describe plasmids as 'avoiding' the trade-off (which they do only in the abstract, and in the discussion L277 so this is quite a minor comment). I agree with the authors' overall description and interpretation of their experimental results, but see this as the plasmids following the trade-off, or evolving along the trade-off, not avoiding it: if most plasmids are close to the intermediate peak evidenced here, this rather suggests that they are constrained by the trade-off, and evolved towards conjugation efficiencies values at which vertical and horizontal transmission trade-off with each other, explaining why higher conjugation efficiencies are not commonly observed.

- Finally, I struggled to follow what experiments were done in some parts of the results, in particular with which plasmids each experiment was done. Is it always the full set of plasmids described in the methods, or were some specific plasmids picked? It might be worth renaming or numbering the plasmids for easier reference.
For instance, starting L122, I got confused with the various references to plasmids / strains used: over 25% of pathogens, then 40 transconjugants, then 27 pathogens (are these 27 the 25%?). Or in L191 and Fig 3B, which were the plasmids used?
The information on plasmids used could also be made clearer in the figures. For instance in Fig 2, the use of colors to indicate growth medium is not necessary as this is already indicated by the different subplots; maybe color, or shape, could indicate each plasmid used, so the reader can understand which of the variation plotted is due to different plasmids conjugating, and which variation is just replicate measurements, which is unclear now.

- Minor comments / points to clarify

L82, donor to any 'non-daughter' recipient (or some similar wording, or else it includes vertical transfer

L83 "they" = plasmid-carrying cells?

L110, I don't understand "serves as an effective differentiator"

L145-147 either some stats are missing, or it is unclear to me what's being tested - can the authors explicitly describe the statistical model(s in the text

L199: where are the data on total cell density? The 'density' in Fig 3C is a distribution, not cell density values?
I also struggled with Fig 3 more generally. I'm not sure where the density plot in Fig 3C comes from, and Fig 3D meaning wasn't clear to me.

L202: can the authors back this with a statistical test? Also, could they do a similar analysis with MOB groups instead of Inc groups?

L227, this isn't a very informative title, and could apply to most paragraphs of the paper

Paragraph starting L245: do the authors suggest that this is adaptive? Or a side-effect of less regulated expression?

L291 adaptive response of whom?

Reviewer #3:

The authors present data showing that conjugation rate is positively correlated with plasmid burden whereby very high conjugation rates result in increased cost of plasmid carriage. The authors go on to show that intermediate conjugation rates, which impose low cost on their hosts, allows the plasmid to spread to higher proportions within populations. RNA sequencing shows that increase transcription of tra genes occurs in strains with higher conjugation rates and is associated with increased cost of carriage in nutrient poor environmental. The paper is well written, the data is very clearly presented, and a lot of consideration has gone into experimental design and analysis. The results will be of interest anyone investigating HGT and particularly plasmid biology. This work contributes to our understanding of plasmid persistence and conjugative transfer. I only have a few minor comments about the manuscript and overall, I think the manuscript is of very high quality.

Throughout the manuscript growth defect due to plasmid carriage is plotted as a positive value (1-(u_p+/u_p-)), to me this is unintuitive and I would expect costs to be expressed as a negative value, which is far more common. What is the justification to expressing costs of plasmid carriage as a positive value?

I would be interested to see discussion by the authors about how positive selection may interact with the trade-off between conjugation rate and cost.

Figure 4, the authors show positive correlation between transcript level and conjugation rate, but what are the significance values of these correlations.

Presumably the benefit of horizontal transfer is dependent upon the frequency of the plasmid within the population - do you see differential benefits of high conjugation rates depending on the proportion of plasmid free recipients within the population?

Reviewer comments and Author's responses

We thank the reviewers for their time and thoughtful comments. Below, we address each comment individually. Responses and changes in the text have been marked in blue. Please note that Supplementary Figures have been changed to Appendix Figures.

Reviewer 1

In this study, Bethke et al. investigate how the trade-off between vertical and horizontal modes of plasmid transfer impact plasmid fitness. This is quite an interesting topic which has been discussed a lot but had not been property assessed until now. This work demonstrates that this trade-off is conserved for multiple wild type plasmids, but that it is only evident from a given threshold of conjugation efficiency (a quite high conjugation rate, actually). This result has important implications for understanding how plasmids are able to survive in bacterial populations. Moreover, the authors show that the expression levels of conjugation genes do in fact correlate with conjugation efficiency, and ultimately with plasmid fitness effects (at least once passed the conjugation threshold), and propose that expression levels could therefore be used to predict these phenotypes. In addition, the authors also used mathematical models that generally agree with their experimental results, in order to generalize their findings. In general, I think that this is an interesting work and would represent an important contribution to the field. I present some minor comments below:

We appreciate the favorable review and attention to the nuance our study provides over previous discussion of the tradeoff.

Some of the key experiments of this study are obviously the conjugation assays. If I understood correctly, the authors conjugated the AMR plasmids from the wild-type enterobacteria to MG1655, and then they calculated the transfer efficiencies between MG1655. I was not able to find the details of the donor and recipient strains (how did the authors differentiate between donor and recipient MG1655, for example) in the methods section. Maybe the authors could provide a little more detail about it?

Yes, this interpretation is correct. Some details for AMR plasmid donors and the MG1655 recipient (DA28102) were included in the "Growth Media" section of Materials and Methods and Appendix Table 1. However, this information should be clarified in the main text and we thank the reviewer for bringing this oversight to our attention. Furthermore, the MG1655 recipient to DA28102 donors was not indicated. We now update the text to include:

> In previous work, over 25% of the pathogen isolates were able to readily transfer their beta-lactam resistance to a susceptible MG1655 E. coli recipient (strain DA28102, Appendix Table 1)
>
> Strain fAYC002 was used as a plasmid-free MG1655 E. coli recipient to maintain plasmid-host compatibility with DA28102 donors (Appendix Table 1)(Chen et al, 2014).
>
> Kanamycin (Kan, 50 μg/mL) was added to select for MG1655 E. coli strain fAYC002.
>
> MG1655 strains DA28102 and fAYC002 served as plasmid donor and recipient, respectively, in all experiments except those in Appendix Figure 7.

This is just a suggestion: In our experience sometimes the area under the growth curve provide a better proxy for fitness than the maximum growth rate. Have you tried running your analyses using the AUC instead of Vmax?

We did not run our analysis with AUC. Using growth rates as the basis to determine growth effects of plasmids allows us to incorporate these effects in our subsequent modelling analysis. That being said, data in

the literature indicate a general agreement among AUC, max growth rate, and other measures of growth curves (Alonso-del Valle *et al*, 2021; Sprouffske & Wagner, 2016). In light of the reviewer's comment, we checked the correlation between AUC and growth rate; we found a strong linear correlation between the two, consistent with the literature. We included this new analysis as Appendix Figure 2 in the revision.

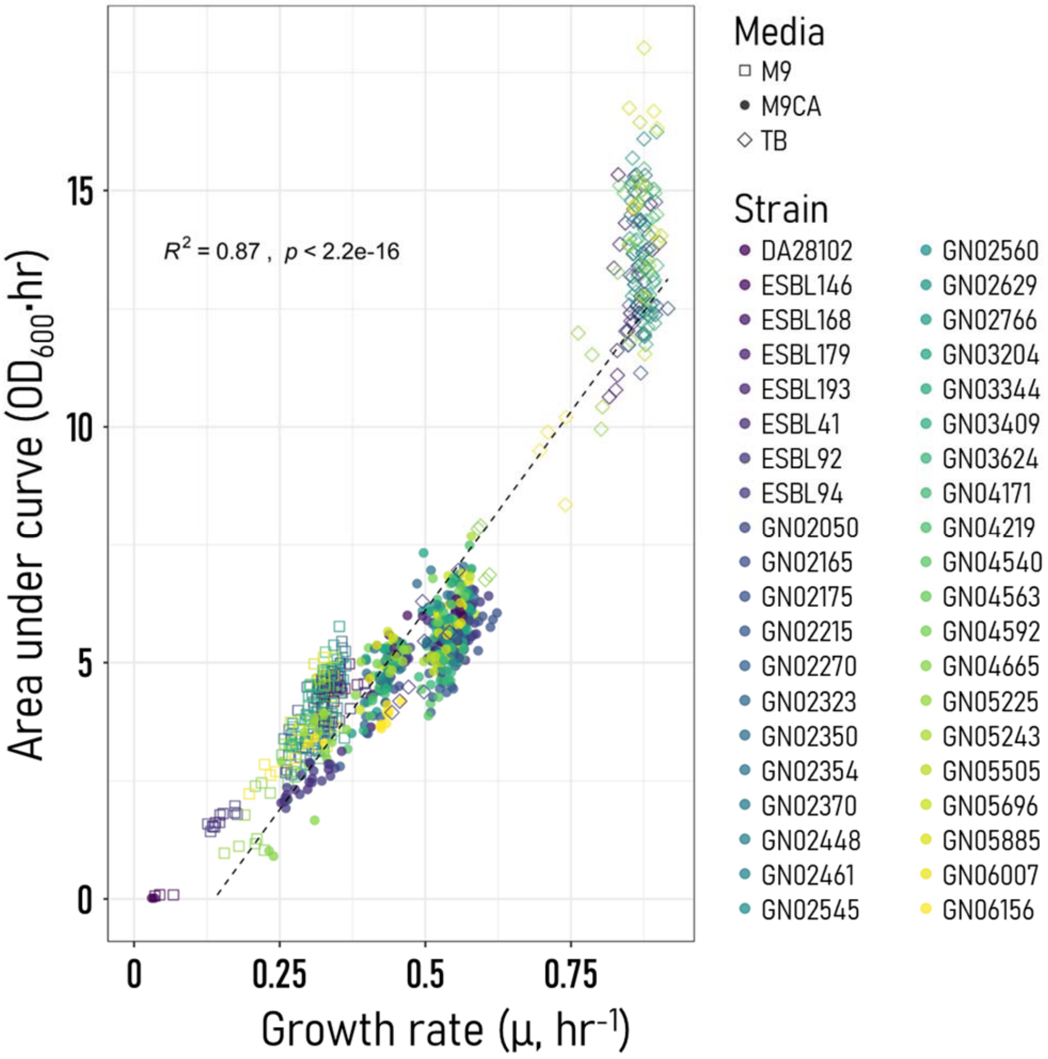

Appendix Figure 2. Correlation between area under curve and specific growth rate. Specific growth rates (μ, hr-1) and areas under growth curves (OD600•hr) are shown from all growth curves collected (n = 881). Growth data include biological replicates in M9, M9CA, and TB media with and without 100 Carb (100 μg/mL) and Cm (50 μg/mL) antibiotics. Growth rates were calculated as in Appendix Figure 1. Areas under growth curves were calculated from blank subtracted and weighted moving average smoothed data.

I think it the term "pathogen plasmid" may be a bit less accurate than maybe "AMR plasmid" or "resistance plasmid", since the plasmid is selected for the AMR mechanism.

We agree that "AMR plasmid" better emphasizes not only how the plasmids were selected, but also plasmids' role in the spread of resistance. This phrasing has been adjusted throughout the text.

Supp figure 3. Plasmid size could be multiplied by plasmid copy number (inferred from the

plasmid/chromosome coverage data from genome sequences) to check if the total extra DNA content correlates with plasmid growth effect, as a possible additional test.

We thank the reviewer for their suggestion and agree this method is a better representation of plasmid DNA's effect on growth effect. We have replaced the previous figures with a single figure incorporating our transconjugant sequencing data and estimated copy number. A caveat of this new analysis is that sequencing samples were from LB culture, which may affect plasmid copy number. This has been indicated in the figure legend.

Relative to the previous analysis, there is a stronger but still weak correlation between estimated total plasmid DNA and growth effect. We included this new analysis as Appendix Figure 6 in the revision.

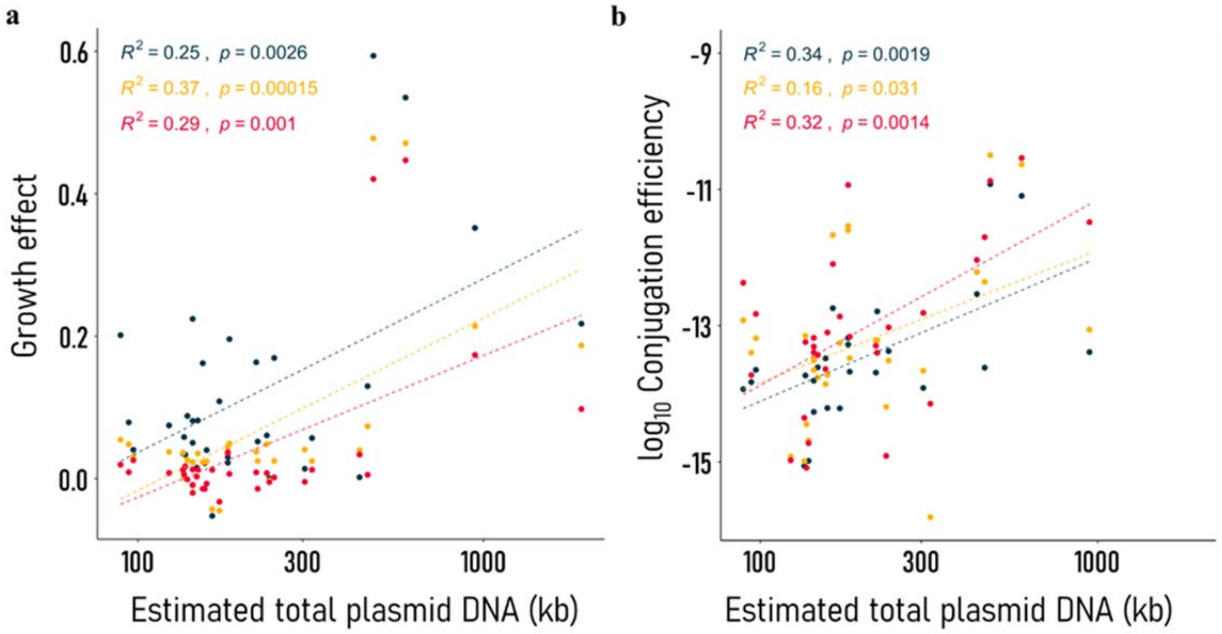

Appendix Figure 6. Correlations with estimated total plasmid DNA. Total plasmid DNA was estimated from sequence length and average sequence coverage relative to the bacterial chromosome. Cultures for sequencing were grown in LB media, which may affect plasmid copy number. (a) The relationship between estimated total plasmid DNA and plasmid growth effect. Average growth effects (n = 3-5 biological replicates) are shown for M9 (blue), M9CA (yellow), and TB (red) media. (b) The relationship between estimated total plasmid DNA and conjugation efficiency. Average conjugation efficiencies (n = 3-6 technical replicates, 1-2 biological replicates) are shown for each media condition.

Define the dashed line in Supp Fig. 6

We have now defined the dashed lines as trendlines between pathogen and MG1655 conjugation efficiencies. See the updated Appendix Figure 7 legend:

Reviewer 2

In this study, the authors measure conjugation cost and study growth-conjugation trade-offs across 40 plasmids from clinical E. coli. They present evidence that conjugation becomes costly at high rates; and that most plasmids transfer at rates below this level. They also show that the cost of conjugation on bacterial growth is stronger in poor medium, and link this cost to transfer gene expression. The idea of a trade-off is not novel, but this study provides a well-executed and interesting, in-depth characterisation of this trade-off across clinical plasmids, with new results on the molecular causes for the cost of conjugation. I have no major criticisms, but some divergences in interpretation with the authors, and a few minor comments to improve clarity.

We appreciate the favorable review and attention to detail found in the alternative interpretations and comments to improve clarity.

- first, I am not convinced by the use of the word 'threshold' to describe the increase in cost only at high conjugation rates. Does the cost still exhibit such visible threshold if conjugation rates are plotted in linear scale? I don't find it surprising, or striking, that such threshold appears to be present when conjugation rates are on a log scale while the effect on growth is effectively a linear measure. I agree with the authors that conjugation rates are usually presented with a log scale, because they can be very small indeed, but not detecting any significant difference between the costs conferred by a 10000th and a 100000th of the maximal transfer efficiency (which is around 10-9 to 10-8) across noisy biological systems is only to be expected.

The reviewer raises a good point about log-linear relationships. As mentioned, conjugation rates are usually presented in log scale as linear scales render the data difficult to interpret (see Figure below).

Indeed, it is precisely our point that conjugation can vary over orders of magnitude without a change in growth effect, suggesting plasmids can be made to persist much better without much effect on fitness. No significant change in growth effect was found up to a 10th or 100th of the maximal conjugation efficiency, depending on media conditions. Until these experiments were performed, there was no a priori basis to expect this result. The conjugation efficiency thresholds in our analysis are not arbitrary; they are determined by fitting using a linear-threshold equation.

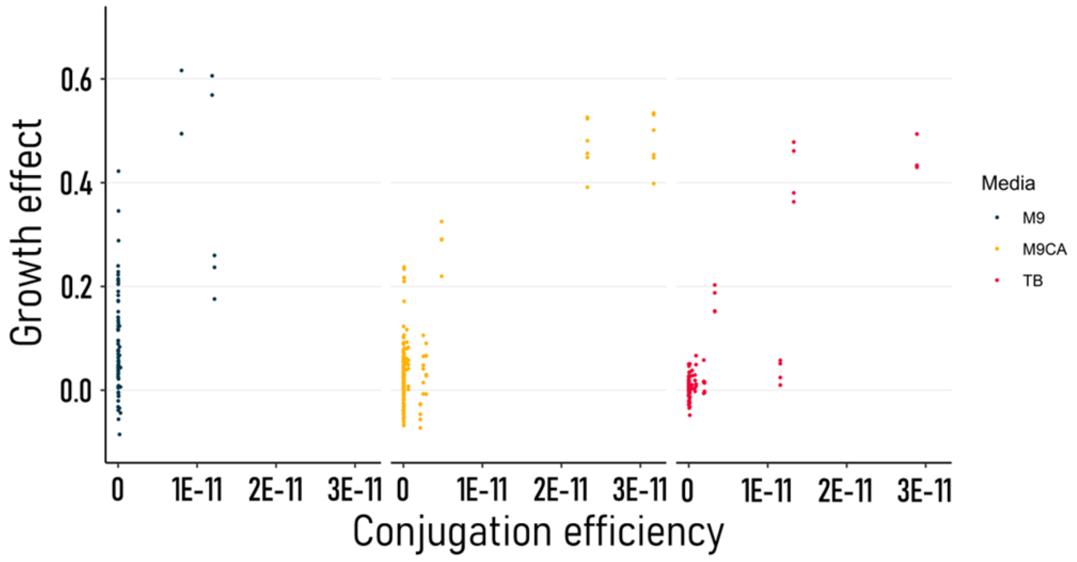

- I also don't agree with the way the authors describe plasmids as 'avoiding' the trade-off (which they do only in the abstract, and in the discussion L277 so this is quite a minor comment). I agree with the authors' overall description and interpretation of their experimental results, but see this as the plasmids following the trade-off, or evolving along the trade-off, not avoiding it: if most plasmids are close to the intermediate peak evidenced here, this rather suggests that they are constrained by the trade-off, and evolved towards conjugation efficiencies values at which vertical and horizontal transmission trade-off with each other, explaining why higher conjugation efficiencies are not commonly observed.

We agree with the reviewer and have adjusted our wording throughout to better adhere to this interpretation.

- Finally, I struggled to follow what experiments were done in some parts of the results, in particular with which plasmids each experiment was done. Is it always the full set of plasmids described in the methods, or were some specific plasmids picked? It might be worth renaming or numbering the plasmids for easier reference.

For instance, starting L122, I got confused with the various references to plasmids / strains used: over 25% of pathogens, then 40 transconjugants, then 27 pathogens (are these 27 the 25%?). Or in L191 and Fig 3B, which were the plasmids used?

In most cases, as many plasmids as possible were used. Not all plasmids transferred at sufficient efficiency to generate transconjugants under every condition, and those are not displayed. This information is included in Appendix Table 2. Resources are limited for certain experiments (e.g. RNAseq, long term) so a select group of plasmids were chosen for diverse Inc, growth effects, conjugation efficiencies:

> RNAseq: Gene expression was measured for MG1655 *E. coli* transconjugants ESBL41T, ESBL92T, GN02560T, GN05243T, and pCU1T carrying pathogen plasmids of IncN/F, IncF/I, IncF/Col, IncB/O/K/Z, and IncN groups, a wide range of conjugation efficiencies, and varied growth effects in both TB and M9 media.

We have updated the former L122 and L191 for clarity:

Former L122: "In previous work, over 25% of the pathogen isolates were able to readily transfer their beta-lactam resistance to a susceptible MG1655 *E. coli* recipient (Supplementary Table 1)(22). The resulting 40 MG1655 transconjugants eliminate host effects, but allow general comparison of the pathogen plasmids."

> **New:** In previous work, we discovered 35 of 143 (~25%) E. coli pathogens were able to readily transfer their beta-lactam resistance to a susceptible MG1655 E. coli recipient (strain DA28102, Appendix Table 1)(Bethke et al, 2020). With additional screening we assembled the collection of 40 MG1655 transconjugants used in this study, each carrying a unique set of pathogen plasmids. A consistent MG1655 host enables general comparison of the pathogen plasmids, but excludes analysis of host effects.

Former L190: "We tested these consequences of a conjugation-growth tradeoff experimentally with pathogen plasmids spanning a wide range of conjugation efficiencies (Figure 3B). For each plasmid, we recreated the starting conditions of the simulation, passaged the mixture of plasmid-carrying and plasmid-free MG1655 E. coli daily for 3 days, then measured abundance and plasmid-carrying fraction.

> **New Fig. 3B legend:** Strains tested include 41T, 92T, 94T, 2350T, 2629T, 3204T, 4219T, 4563T, 4592T, and 5696T. Conjugation efficiencies for strains 2350T and 3204T fell outside the measurable range in M9 media.

The information on plasmids used could also be made clearer in the figures. For instance in Fig 2, the use of colors to indicate growth medium is not necessary as this is already indicated by the different subplots; maybe color, or shape, could indicate each plasmid used, so the reader can understand which of the variation plotted is due to different plasmids conjugating, and which variation is just replicate measurements, which is unclear now.

We agree that color-coding of the experimental conditions in this figure is unnecessary. Data from the three conditions were used for other analysis (e.g., Appendix Figure 1, Appendix Figure 3, Appendix Figure 6), where color coding becomes necessary.

Also, we agree that the ability to distinguish among each plasmid would be ideal. Considering the density and number of points plotted, however, distinguishing shapes or colors may make the figure confusing. We note that the detailed information regarding strains/plasmids and plasmid parameters, under all three conditions, is also presented in Appendix Table 2. Each data point in Figure 2 is the average from multiple replicates, as explained in the Figure caption.

- Minor comments / points to clarify

L82, donor to any 'non-daughter' recipient (or some similar wording), or else it includes vertical transfer

We appreciate the suggestion. We have now specified horizontal gene transfer as transfer "outside of cell division" to exclude vertical transfer.

L83 "they" = plasmid-carrying cells?

Yes; this was unclear, thank you. We have updated the text accordingly.

L110, I don't understand "serves as an effective differentiator"

We have reworded this section as follows:

> We find a standard MG1655 E. coli host can support a wide range of conjugation efficiencies with only minor growth effects, placing most plasmids below the threshold. For this majority of plasmids with similar growth effects, conjugation efficiency then serves to differentiate and predict plasmid fitness.

L145-147 either some stats are missing, or it is unclear to me what's being tested - can the authors explicitly describe the statistical model(s) in the text

We have updated the text for clarity, separating statements of significance from conclusions drawn from correlations:

> There was a significant effect of media environment on growth effect per strain [$F(78, 398) = 6.27$, $P < 0.0001$, ANOVA] and conjugation efficiency [$F(1, 25) = 13.7$, $P = 0.001$, ANOVA]. Generally, growth effects decreased and conjugation efficiency increased with increasing nutrient content (Appendix Figure 6).

L199: where are the data on total cell density? The 'density' in Fig 3C is a distribution, not cell density values? I also struggled with Fig 3 more generally. I'm not sure where the density plot in Fig 3C comes from, and Fig 3D meaning wasn't clear to me.

We regret not being more specific in the text. The data on total cell density is in Figure 3B, indicated by grey points. These are linked to blue or red points, depending on the media environment, indicating the plasmid abundance for that culture. We've now included a reference to Figure 3B in the statement about total cell density and changed the y axis label on Figure 3C to "probability density" for clarity.

Figure 3C is the probability density of conjugation efficiencies across media environments. This reflects where most plasmids fall along the conjugation efficiency axis. In Figure 3D, we relate the distribution in 3C (via summary statistics) to the fitness data in 3B. We find the plasmid(s) that achieve the highest abundance and/or fraction in 3B have conjugation efficiencies that would be considered common from 3C. This result supports the generality of the conjugation-growth tradeoff and helps explain why different plasmids appear to converge upon similar conjugation efficiencies.

L202: can the authors back this with a statistical test? Also, could they do a similar analysis with MOB groups instead of Inc groups?

As this result only played a minor role in our overall findings, we have decided to remove this figure and replace it with the area under the curve growth analysis requested by Reviewer 1. We direct the reviewer to our previous work on the topic, with statistical analysis:

> Bethke JH, Davidovich A, Cheng L, Lopatkin AJ, Song W, Thaden JT, Fowler VG, Xiao M & You L (2020) Environmental and genetic determinants of plasmid mobility in pathogenic Escherichia coli. Sci Adv 6

L227, this isn't a very informative title, and could apply to most paragraphs of the paper

We agree and have replaced the title with "The relative cost of horizontal transfer within a host" to better match the section content.

Paragraph starting L245: do the authors suggest that this is adaptive? Or a side-effect of less regulated expression?

Indeed, we speculate that increased resources being funneled into HGT under nutrient stress could be adaptive. Also, as suggested by the reviewer, it could be the result of less regulated expression. We have revised the text to incorporate the suggestion.

> This seemingly parasitic behavior exacerbates the growth tradeoff, but may also represent an adaptive host response to transient stress or less regulated expression (Rodríguez-Beltrán et al, 2021).

L291 adaptive response of whom?

In this instance we meant the host, but it was not clear. We have now updated the sentence to be more specific (see above).

Reviewer 3

The authors present data showing that conjugation rate is positively correlated with plasmid burden whereby very high conjugation rates result in increased cost of plasmid carriage. The authors go on to show that intermediate conjugation rates, which impose low cost on their hosts, allows the plasmid to spread to higher proportions within populations. RNA sequencing shows that increase transcription of tra genes occurs in strains with higher conjugation rates and is associated with increased cost of carriage in nutrient poor environmental. The paper is well written, the data is very clearly presented, and a lot of consideration has gone into experimental design and analysis. The results will be of interest anyone investigating HGT and particularly plasmid biology. This work contributes to our understanding of plasmid persistence and conjugative transfer. I only have a few minor comments about the manuscript and overall, I think the manuscript is of very high quality.

We appreciate the favorable review and recognition for experimental design and analysis. We're happy to hear the text and figures were clear.

Throughout the manuscript growth defect due to plasmid carriage is plotted as a positive value (1-(u_p+/u_p-)), to me this is unintuitive and I would expect costs to be expressed as a negative value, which is far more common. What is the justification to expressing costs of plasmid carriage as a positive value?

From discussions with our colleagues, it was suggested we formulate plasmid growth effect in the current way to center the plots around 0 and emphasize greater values as greater growth burdens. We do understand the intuition behind the reviewer's suggestion, and, if the reviewer feels strongly about this, we have no issue adjusting the text and figures to the alternative. It would not change the conclusions.

I would be interested to see discussion by the authors about how positive selection may interact with the trade-off between conjugation rate and cost.

Thanks for the suggestion. Positive selection on a plasmid (e.g. by antibiotic selection) reduces the cost of the plasmid or makes it beneficial. If the selection is sufficiently strong, the plasmid will always persist, regardless of its transfer rate (even if the rate is zero, for a non-mobilizable plasmid). As such, a strong positive selection will reduce or eliminate the impact of the tradeoff.

We have now added a brief discussion of how positive selection affects the tradeoff.

Figure 4, the authors show positive correlation between transcript level and conjugation rate, but what are the significance values of these correlations.

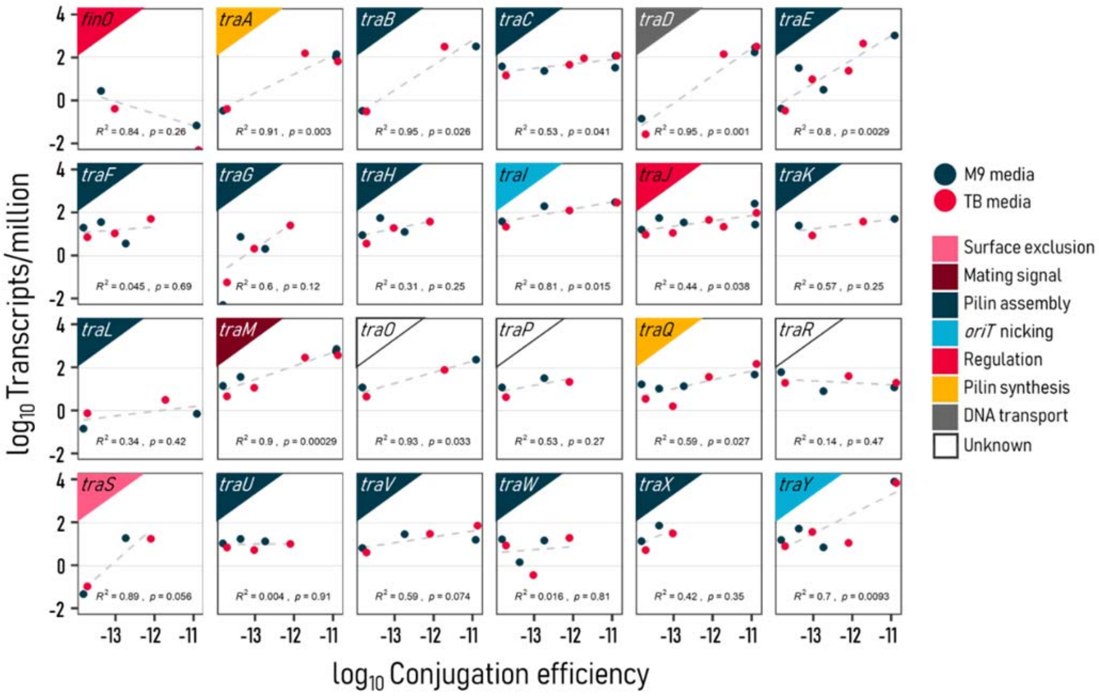

We have now updated Figure 4 to include the significance values of these correlations.

Presumably the benefit of horizontal transfer is dependent upon the frequency of the plasmid within the population - do you see differential benefits of high conjugation rates depending on the proportion of plasmid free recipients within the population?

Indeed, the benefit of horizontal transfer is thought to increase as the frequency of recipients increases and vice versa (Turner *et al*, 1998; Dimitriu *et al*, 2021). Without a constant influx of new recipients into the system, the proportion of plasmid-free recipients will change over time in an experiment and should approach an equilibrium.

This equilibrium point was our primary interest as a measure of plasmid fitness. All of our experiments began with an approximate 1:1 ratio between donors and recipients and were otherwise left to proceed, sometimes with dilution (as in Figures 3A and 3B). We therefore cannot comment on the effect of recipient proportion as if it were a constant.

Thank you for sending us your revised manuscript. We have now heard back from reviewer #2 who was asked to evaluate your revised study. As you will see below, the reviewer is satisfied with the modifications made and supports publication. They only list a few minor issues, which we would ask you to fix in a final round of revision. We would also ask you to address the editorial issues listed below.

Reviewer #2:

The manuscript is overall much clearer and very nuanced, and I'm happy for it to be published, with only a few remaining comments:

- L145 what does plasmid-host compatibility mean

- L151-153 I still don't understand the stats. What are the degrees of freedom here? If the same factor tested is the media environment for both growth and conjugation, why different DF?

- I think this still needs more detail and logic on the strains and plasmids used.
E.g. for fAYC002, it is not clear what it exactly consists in: It is not MG1655 itself if it has been modified, but should be written as MG1655 [insert relevant genotype here].
I don't think the reader should have to go get the cited reference to at least get an idea of how modified the strain was. (I did eventually manage to find that in the supplementary material of the cited Chen et al).

And which transconjugants were used is also still a bit confusing. How can we know which of the plasmids from the donors were transferred, was there some curation step so that the same plasmid or mix of plasmids wasn't characterised twice?

Sorry to be a pain about this, but I've spent too much time in my life trying to go back paper trails to figure out the relevant strain genotype used in experiments. Even if it doesn't appear relevant here, we might discover in a few years that whichever locus the kanamycin resistance gene is inserted at in fAYC002 interferes with conjugation!

- L224: of x, y x groups - respectively -?

11th Dec 2022

Manuscript Number: MSB-2022-11300R
Title: Vertical and horizontal gene transfer tradeoffs direct plasmid fitness

Dear Dr. Polychronidou,

Please find our responses to the editorial issues below.

Thank you for sending us your revised manuscript. We have now heard back from reviewer #2 who was asked to evaluate your revised study. As you will see below, the reviewer is satisfied with the modifications made and supports publication. They only list a few minor issues, which we would ask you to fix in a final round of revision. We would also ask you to address the editorial issues listed below.

- In the Data Availability section please refer to the specific GitHub repositories related to this study (currently the link leads to the lab GitHub page).

The full link to the specific GitHub repository (https://github.com/youlab/PlasmidTradeoff_Bethke) is now included. In addition, our RNAseq submission to GEO has been processed and given the accession number GSE219270.

- Appendix Figures and Tables need to be renamed to Appendix Figure S1-S8 and Appendix Table S1-S2 and called out accordingly (instead of Appendix Figures 1-8 and Appendix Tables 1-2, which is incorrect). Please make sure that the file names are corrected both in the main text (callouts) and in the Appendix file itself.

We have added the "S" to Appendix Figure and Table references, in both the Appendix and Main Text.

- Please include page numbers in the Appendix Table of Contents.

Page numbers for Appendix Figures S1 to S8 (pgs. 2-9) and Tables S1 to S2 (pgs. 10-14) have been added to the Appendix Table of Contents.

- Our data editors have noticed some unclear or missing information in the figure legends, please see the attached .doc file. Please make all requested text changes using the attached file and *keeping the "track changes" mode* so that we can easily access the edits made.

We have clarified that the replicates were biological, specified the range for n, and removed mention of standard deviation for Figure 5A. Variability is instead shown by data points overlaid on the bars.

Please resubmit your revised manuscript online, with a covering letter listing amendments and responses to each point raised by the referees. Please resubmit the paper **within one month** and ideally as soon as possible. If we do not receive the revised manuscript within this time period, the file might be closed and any subsequent resubmission would be treated as a new manuscript. Please use the Manuscript Number (above) in all correspondence.

Click on the link below to submit your revised paper.

https://msb.msubmit.net/cgi-bin/main.plex

Thank you for submitting this paper to Molecular Systems Biology.

Kind regards,

Reviewer comments and Author's responses

Reviewer #2:

The manuscript is overall much clearer and very nuanced, and I'm happy for it to be published, with only a few remaining comments:

We are pleased to see the revisions improved the manuscript and have addressed the remaining comments below.

- L145 what does plasmid-host compatibility mean

Plasmids differ in their host range and behavior within the same host. Even within a species, we see significant variability in conjugation efficiency for the same plasmid (Appendix Figure S7). We paired MG1655 donor strain DA28102 with the MG1655 fAYC002 recipient for consistency. We have simplified the text to:

> Strain fAYC002 was used as a plasmid-free MG1655 *E. coli* recipient consistent with DA28102 donors (Appendix Table 1)(Chen et al, 2014).

- L151-153 I still don't understand the stats. What are the degrees of freedom here? If the same factor tested is the media environment for both growth and conjugation, why different DF?

Upon further review, conjugation efficiencies were only compared between M9 and TB media, without separating by strain. This was to show a general effect between the most distinct media environments. Strain level comparisons are shown in Appendix Figure S5. We now updated the text to reflect this:

> There was a significant effect of media environment on growth effect per strain [$F(78, 398) = 6.27$, $P < 0.0001$, ANOVA] and conjugation efficiency [$F(1, 52) = 4.92$, $P = 0.031$, ANOVA], specifically between TB and M9 media.

- I think this still needs more detail and logic on the strains and plasmids used.
E.g. for fAYC002, it is not clear what it exactly consists in: It is not MG1655 itself if it has been modified, but should be written as MG1655 [insert relevant genotype here].
I don't think the reader should have to go get the cited reference to at least get an idea of how modified the strain was. (I did eventually manage to find that in the supplementary material of the cited Chen et al).

We agree the information should be readily available. We have now included the relevant genotypes for each strain in the Materials and Methods:

> MG1655 E. coli strain DA28102 has the following genotype: F- λ- ilvG- rfb-50 rpb-1 galK::cat-J23101-mTagBFP2. Similarly, the genotype of MG1655 E. coli strain fAYC002 is as follows: PRO ΔcsgA ompR234.

And which transconjugants were used is also still a bit confusing. How can we know which of the plasmids from the donors were transferred, was there some curation step so that the same plasmid or mix of plasmids wasn't characterised twice?

Yes, sequencing was done to find the exact plasmid composition and estimated copy number for each DA28102 transconjugant.

"Follow-up sequencing was performed to determine the plasmid composition of transconjugants. From the 27 pathogen donors carrying multiple plasmids, only 4 (15%) MG1655 transconjugants received and maintained two plasmids (Appendix Table 1). All other transconjugants carry a single, conjugative plasmid from the pathogen donor."

In the majority of cases, only a single plasmid was transferred. Donors were never mixed in the same conjugation experiment. This leaves no ambiguity in subsequent transfers from DA28102 to fAYC002. DA28102 strains carrying 2 plasmids are labeled in Appendix Figure S3 and Appendix Table S1.

To clear up confusion about our results, we specify in the methods:

"MG1655 strains DA28102 and fAYC002 served as plasmid donor and recipient, respectively, in all experiments except those in Appendix Figure 7."

Sorry to be a pain about this, but I've spent too much time in my life trying to go back paper trails to figure out the relevant strain genotype used in experiments. Even if it doesn't appear relevant here, we might discover in a few years that whichever locus the kanamycin resistance gene is inserted at in fAYC002 interferes with conjugation!

- L224: of x, y x groups - respectively -?

Yes, thank you. We had ordered the list of plasmids to match the strains and now include respectively to indicate as such:

Gene expression was measured for MG1655 E. coli transconjugants ESBL41T, ESBL92T, GN02560T, GN05243T, and pCU1T carrying AMR plasmids of IncN/F, IncF/I, IncF/Col, IncB/O/K/Z, and IncN groups, respectively, a wide range of conjugation efficiencies, and varied growth effects in both TB and M9 media.

2nd Revision - Editorial Decision 12th Dec 2022

Thank you again for sending us your revised manuscript. We are now satisfied with the modifications made and I am pleased to inform you that your paper has been accepted for publication.

EMBO Press Author Checklist

Corresponding Author Name: Lingchong You	
Journal Submitted to: Molecular Systems Biology	
Manuscript Number: MSB-2022-11300R	

USEFUL LINKS FOR COMPLETING THIS FORM
The EMBO Journal - Author Guidelines
EMBO Reports - Author Guidelines
Molecular Systems Biology - Author Guidelines
EMBO Molecular Medicine - Author Guidelines

Reporting Checklist for Life Science Articles (updated January
This checklist is adapted from Materials Design Analysis Reporting (MDAR) Checklist for Authors. MDAR establishes a minimum set of requirements in transparent reporting in the life sciences (see Statement of Task: 10.31222/osf.io/9sm4x). Please follow the journal's guidelines in preparing your
Please note that a copy of this checklist will be published alongside your article.

Abridged guidelines for figures
1. Data
The data shown in figures should satisfy the following conditions:
- the data were obtained and processed according to the field's best practice and are presented to reflect the results of the experiments in an accurate and unbiased manner.
- ideally, figure panels should include only measurements that are directly comparable to each other and obtained with the same assay.
- plots include clearly labeled error bars for independent experiments and sample sizes. Unless justified, error bars should not be shown for technical
- if n<5, the individual data points from each experiment should be plotted. Any statistical test employed should be justified.
- Source Data should be included to report the data underlying figures according to the guidelines set out in the authorship guidelines on Data

2. Captions
Each figure caption should contain the following information, for each panel where they are relevant:
- a specification of the experimental system investigated (eg cell line, species name).
- the assay(s) and method(s) used to carry out the reported observations and measurements.
- an explicit mention of the biological and chemical entity(ies) that are being measured.
- an explicit mention of the biological and chemical entity(ies) that are altered/varied/perturbed in a controlled manner.
- the exact sample size (n) for each experimental group/condition, given as a number, not a range;
- a description of the sample collection allowing the reader to understand whether the samples represent technical or biological replicates (including how many animals, litters, cultures, etc.).
- a statement of how many times the experiment shown was independently replicated in the laboratory.
- definitions of statistical methods and measures:
  - common tests, such as t-test (please specify whether paired vs. unpaired), simple χ2 tests, Wilcoxon and Mann-Whitney tests, can be unambiguously identified by name only, but more complex techniques should be described in the methods section;
  - are tests one-sided or two-sided?
  - are there adjustments for multiple comparisons?
  - exact statistical test results, e.g., P values = x but not P values < x;
  - definition of 'center values' as median or average;
  - definition of error bars as s.d. or s.e.m.

Please complete ALL of the questions below.
Select "Not Applicable" only when the requested information is not relevant for your study.

Materials

Newly Created Materials	Information included in the manuscript?	In which section is the information available? (Reagents and Tools Table, Materials and Methods, Figures, Data Availability Section)
New materials and reagents need to be available; do any restrictions apply?	Not Applicable	

Antibodies	Information included in the manuscript?	In which section is the information available? (Reagents and Tools Table, Materials and Methods, Figures, Data Availability Section)
For **antibodies** provide the following information:<br>- Commercial antibodies: RRID (if possible) or supplier name, catalogue number and or/clone number<br>- Non-commercial: RRID or citation	Not Applicable	

DNA and RNA sequences	Information included in the manuscript?	In which section is the information available? (Reagents and Tools Table, Materials and Methods, Figures, Data Availability Section)
**Short novel DNA or RNA including primers, probes**: provide the sequences.	Not Applicable	

Cell materials	Information included in the manuscript?	In which section is the information available? (Reagents and Tools Table, Materials and Methods, Figures, Data Availability Section)
**Cell lines:** Provide species information, strain. Provide accession number in repository **OR** supplier name, catalog number, clone number, and/**OR** RRID.	Not Applicable	
**Primary cultures:** Provide species, strain, sex of origin, genetic modification status.	Not Applicable	
Report if the cell lines were recently **authenticated** (e.g., by STR profiling) and tested for mycoplasma contamination.	Not Applicable	

Experimental animals	Information included in the manuscript?	In which section is the information available? (Reagents and Tools Table, Materials and Methods, Figures, Data Availability Section)
**Laboratory animals or Model organisms:** Provide species, strain, sex, age, genetic modification status. Provide accession number in repository **OR** supplier name, catalog number, clone number, **OR** RRID.	Not Applicable	
**Animal observed in or captured from the field:** Provide species, sex, and age where possible**.**	Not Applicable	
Please detail **housing and husbandry conditions**.	Not Applicable	

Plants and microbes	Information included in the manuscript?	In which section is the information available? (Reagents and Tools Table, Materials and Methods, Figures, Data Availability Section)
**Plants:** provide species and strain, ecotype and cultivar where relevant, unique accession number if available, and source (including location for collected wild specimens).	Not Applicable	
**Microbes:** provide species and strain, unique accession number if available, and source.	Yes	Materials and Methods, Figures, Data Availability Section

Human research participants	Information included in the manuscript?	In which section is the information available? (Reagents and Tools Table, Materials and Methods, Figures, Data Availability Section)
If collected and within the bounds of privacy constraints report on age, sex and gender or ethnicity for all study participants.	Not Applicable	

Core facilities	Information included in the manuscript?	In which section is the information available? (Reagents and Tools Table, Materials and Methods, Figures, Data Availability Section)
If your work benefited from core facilities, was their service mentioned in the acknowledgments section?	Yes	Acknowledgements

Design

Study protocol	Information included in the manuscript?	In which section is the information available? (Reagents and Tools Table, Materials and Methods, Figures, Data Availability Section)
If study protocol has been **pre-registered, provide DOI in the manuscript**. For clinical trials, provide the trial registration number **OR** cite DOI.	Not Applicable	
Report the **clinical trial registration number** (at ClinicalTrials.gov or equivalent), where applicable.	Not Applicable	

Laboratory protocol	Information included in the manuscript?	In which section is the information available? (Reagents and Tools Table, Materials and Methods, Figures, Data Availability Section)
Provide DOI OR other citation details if **external detailed step-by-step protocols** are available.	Not Applicable	

Experimental study design and statistics	Information included in the manuscript?	In which section is the information available? (Reagents and Tools Table, Materials and Methods, Figures, Data Availability Section)
Include a statement about **sample size** estimate even if no statistical methods were used.	Yes	Materials and Methods, Figures
Were any steps taken to minimize the effects of subjective bias when allocating animals/samples to treatment (e.g. **randomization procedure**)? If yes, have they been described?	Not Applicable	
Include a statement about **blinding** even if no blinding was done.	Not Applicable	
Describe **inclusion/exclusion criteria** if samples or animals were excluded from the analysis. Were the criteria pre-established?<br><br>If sample or data points were omitted from analysis, report if this was due to attrition or intentional exclusion and provide justification.	Yes	Materials and Methods, Figures
For every figure, are **statistical tests** justified as appropriate? Do the data meet the assumptions of the tests (e.g., normal distribution)? Describe any methods used to assess it. Is there an estimate of variation within each group of data? Is the variance similar between the groups that are being statistically compared?	Yes	Materials and Methods, Figures

Sample definition and in-laboratory replication	Information included in the manuscript?	In which section is the information available? (Reagents and Tools Table, Materials and Methods, Figures, Data Availability Section)
In the figure legends: state number of times the experiment was **replicated** in laboratory.	Yes	Materials and Methods, Figures
In the figure legends: define whether data describe **technical or biological replicates**.	Yes	Materials and Methods, Figures

Ethics

Ethics	Information included in the manuscript?	In which section is the information available? (Reagents and Tools Table, Materials and Methods, Figures, Data Availability Section)
Studies involving **human participants**: State details of **authority granting ethics approval** (IRB or equivalent committee(s), provide reference number for approval.	Not Applicable	
Studies involving **human participants**: Include a statement confirming that **informed consent** was obtained from all subjects and that the experiments conformed to the principles set out in the WMA Declaration of Helsinki and the Department of Health and Human Services Belmont Report.	Not Applicable	
Studies involving **human participants:** For publication of **patient photos**, include a statement confirming that consent to publish was obtained.	Not Applicable	
Studies involving experimental **animals**: State details of **authority granting ethics approva**l (IRB or equivalent committee(s), provide reference number for approval. Include a statement of compliance with ethical regulations.	Not Applicable	
Studies involving **specimen and field samples:** State if relevant **permits** obtained, provide details of authority approving study; if none were required, explain why.	Not Applicable	

Dual Use Research of Concern (DURC)	Information included in the manuscript?	In which section is the information available? (Reagents and Tools Table, Materials and Methods, Figures, Data Availability Section)
Could your study fall under dual use research restrictions? Please check biosecurity documents and list of **select agents and toxins** (CDC): https://www.selectagents.gov/sat/list.htm	Not Applicable	
If you used a select agent, is the security level of the lab appropriate and reported in the manuscript?	Not Applicable	
If a study is subject to dual use research of concern regulations, is the name of the **authority granting approval and reference number** for the regulatory approval provided in the manuscript?	Not Applicable	

Reporting

The MDAR framework recommends adoption of discipline-specific guidelines, established and endorsed through community initiatives. Journals have their own policy about requiring specific guidelines and recommendations to complement MDAR.

Adherence to community standards	Information included in the manuscript?	In which section is the information available? (Reagents and Tools Table, Materials and Methods, Figures, Data Availability Section)
State if relevant guidelines or checklists (e.g., **ICMJE, MIBBI, ARRIVE, PRISMA**) have been followed or provided.	Not Applicable	
For **tumor marker prognostic studies,** we recommend that you follow the **REMARK** reporting guidelines (see link list at top right). See author guidelines, under 'Reporting Guidelines'. Please confirm you have followed these guidelines.	Not Applicable	
For **phase II and III randomized controlled trials**, please refer to the **CONSORT** flow diagram (see link list at top right) and submit the CONSORT checklist (see link list at top right) with your submission. See author guidelines, under 'Reporting Guidelines'. Please confirm you have submitted this list.	Not Applicable	

Data Availability

Data availability	Information included in the manuscript?	In which section is the information available? (Reagents and Tools Table, Materials and Methods, Figures, Data Availability Section)
Have **primary datasets** been deposited according to the journal's guidelines (see 'Data Deposition' section) and the respective accession numbers provided in the Data Availability Section?	Yes	Data Availability Section
Were **human clinical and genomic datasets** deposited in a public access-controlled repository in accordance to ethical obligations to the patients and to the applicable consent agreement?	Not Applicable	
Are **computational models** that are central and integral to a study available without restrictions in a machine-readable form? Were the relevant accession numbers or links provided?	Yes	Materials and Methods
If publicly available data were reused, provide the respective **data citations in the reference list**.	Yes	Materials and Methods, References, Data Availability Section
